# 17β-Estradiol Delivered in Eye Drops: Evidence of Impact on Protein Networks and Associated Biological Processes in the Rat Retina through Quantitative Proteomics

**DOI:** 10.3390/pharmaceutics12020101

**Published:** 2020-01-27

**Authors:** Laszlo Prokai, Khadiza Zaman, Vien Nguyen, Katalin Prokai-Tatrai

**Affiliations:** 1 Department of Pharmacology and Neuroscience, University of North Texas Health Science Center, Fort Worth, TX 76107, USA; Khadiza.Zaman@unthsc.edu (K.Z.); Vien.Nguyen@unthsc.edu (V.N.); Katalin.Prokai@unthsc.edu (K.P.-T.); 2 Institute for Healthy Aging, University of North Texas Health Science Center, Fort Worth, TX 76107, USA

**Keywords:** eye drops, estradiol, female rats, retina, proteomics, label-free quantitation, pathway analysis, biological networks, target engagement, crystallin

## Abstract

To facilitate the development of broad-spectrum retina neuroprotectants that can be delivered through topical dosage forms, this proteomics study focused on analyzing target engagements through the identification of functional protein networks impacted after delivery of 17β-estradiol in eye drops. Specifically, the retinae of ovariectomized Brown Norway rats treated with daily eye drops of 17β-estradiol for three weeks were compared to those of vehicle-treated ovariectomized control animals. We searched the acquired raw data against a composite protein sequence database by using Mascot, as well as employed label-free quantification to detect changes in protein abundances. Our investigation using rigorous validation criteria revealed 331 estrogen-regulated proteins in the rat retina (158 were up-regulated, while 173 were down-regulated by 17β-estradiol delivered in eye drops). Comprehensive pathway analyses indicate that these proteins are relevant overall to nervous system development and function, tissue development, organ development, as well as visual system development and function. We also present 18 protein networks with associated canonical pathways showing the effects of treatments for the detailed analyses of target engagements regarding potential application of estrogens as topically delivered broad-spectrum retina neuroprotectants. Profound impact on crystallins is discussed as one of the plausible neuroprotective mechanisms.

## 1. Introduction

The development of clinical interventions targeting eye diseases with new chemical entities has been burdened by increasing costs and high rate of regulatory failures [1]. Therefore, medical needs unmet by currently marketed ocular drug products have been emerging [2]. For example, preventative, disease-modifying, or adjuvant medications that would thwart or remedy damage to retinal neurons by a group of complex and slow-progressing neurodegenerative eye diseases such as glaucoma, diabetic retinopathy, or age-related macular degeneration have remained elusive [3]. However, it has been recognized that many approved drugs can be repurposed for ocular pharmacotherapy [4,5], or utilized as direct lead compounds to develop ophthalmic pharmaceuticals [6,7,8]. Another challenge is that pathology-associated neuronal damage in the retina exhibits multiple mechanisms of yet to be understood complexity, which calls for polypharmacology (which recognizes the necessity for a single drug to exert effects on multiple targets and disease pathways) as a preventative and/or therapeutic approach [9]. In addition, eye drops have remained the most common route of ophthalmic drug delivery despite their noted limitations [10,11]. Altogether, preferred therapeutic candidates for the purpose would be broad-spectrum retina neuroprotectants [12] that could be delivered even using simple topical applications [10].

The pleiotropic and tissue-specific action of estrogens has been the subject of many important basic and translational studies [13]. Besides their well-researched reproductive function [14], they also impact the central nervous system (CNS) by multiple genomic and non-genomic mechanisms that are often implicated in preventative and therapeutic contexts [15,16]. These include broad-spectrum neuroprotection against the aging of the retina and age-related ocular neurodegenerative diseases [17,18]. In fact, estrogen deficiency has been linked recently to a glaucoma phenotype in mice [19]. Our previous studies have convincingly shown that administration of 17β-estradiol (E_2_) as eye drops can provide therapeutic drug levels in the retina, which was associated with profound protection of retinal ganglion cells and preservation of visual acuity in a rat glaucoma model [20]. However, understanding how the hormone acts in the retina has remained rudimentary, and few estrogen-regulated retinal proteins have been identified to date [21], even relying on early proteomics studies [20,22]. Here, we present an expanded study relying on our previous in vivo experimental design [20], but performed using a newer, more powerful instrument for label-free quantitative proteomic analyses. In particular, we reveal potential therapeutic target engagements through the identification of functional protein networks for future translational studies focused on retina neuroprotection after E_2_ delivery in eye drops.

## 2. Materials and Methods 

### 2.1. Chemicals and Reagents

E_2_, 2-hydroxypropyl-β-cyclodextrin, urea, dithiothreitol, iodoacetamide, ammonium bicarbonate, and formic acid (ACS reagent grade, ≥98%) were purchased from MilliporeSigma (St. Louis, MO, USA). Sequencing-grade trypsin was obtained from Promega (Madison, WI, USA). Water and acetonitrile were Optima^®^ LC/MS grade, and supplied by Thermo Fisher Scientific (Waltham, MA, USA).

### 2.2. Animals and Treatments

All procedures involving animals were reviewed and approved by the Institutional Animal Care and Use Committee at the University of North Texas Health Science Center before the initiation of the studies (approval number: 2018-0028, on 10 July 2018). Five ovariectomized (OVX) Brown Norway rats (Charles Rivers Laboratories, Wilmington, DE, USA) weighing 200–250 g received 10 µL of 17β-estradiol (E_2_) eye drops once daily in both eyes for three weeks [20]. The eye drops contained 0.1% (*w*/*v*) E_2_ in saline vehicle containing 20% (*w*/*v*) 2-hydroxypropyl-β-cyclodextrin. Five OVX control rats received 10 µL of this vehicle as eye drops for the same dosing regimen and duration. After 24 h of the last treatment, they were euthanized, and their eyes were immediately enucleated followed by rapid isolation of the retina. Tissue samples were rinsed with saline and then blotted dry for preparation to label-free shotgun proteomic analyses according to previously published methods [20,23].

### 2.3. Sample Preparation

Each isolated retina was prepared first by incubating in 200 µL 8 M aqueous urea solution for 30 min followed by centrifugation for 5 min at 1400× *g* [20,23]. The supernatant was collected, and protein content was measured using a microplate reader (BioTek Synergy H1 with Take3 plate; Agilent, Santa Clara, CA, USA). An aliquot containing 100 µg protein was taken and the volume was adjusted with 25 mM ammonium bicarbonate to 100 µL. The sample was then reduced with dithiothreitol and carbamidomethylated with iodoacetic amide [20,23]. After 9-fold dilution of the sample with aqueous 25 mM ammonium bicarbonate, trypsin digestion was performed at 37 °C overnight, then the reaction was quenched by acidification with formic acid (1 µL). The solution was desalted by solid-phase extraction (SPE) using 1 mL Sep-Pak™ C18 SPE cartridges (Waters, Milford, MA, USA), and the extract was dried under vacuum (Vacufuge™, Eppendorf AG, Hamburg, Germany) into a 1.5 mL centrifuge tube.

### 2.4. Proteomics Analyses

The digested samples were analyzed using a hybrid ion trap–Orbitrap tandem mass spectrometer (LTQ Velos Orbitrap Pro) coupled to an EASY nLC-1000 nanoflow liquid chromatography system fitted with a 15 cm × 75 μm i.d. EasySpray column packed with 3 µm PepMap C18 particles (Thermo Fisher Scientific, San Jose, CA, USA). Gradient elution was used: Solvent A and solvent B were water and acetonitrile, respectively, and each contained 0.1% (*v*/*v*) formic acid. Samples (100 µg protein) were reconstituted in 100 µL of solvent containing 5% (*v*/*v*) acetonitrile and 0.1% (*v*/*v*) formic acid in water and transferred into 200 µL polypropylene autosampler vials closed with an open-top screw cap and Teflon-lined silicon septum (USA Scientific, Orlando, FL, USA). During a 20 min column equilibration at 5% B, 5 µL of solution was injected while maintaining constant column pressure at 600 bar. The peptides were eluted at 300 nL/min using the following gradient: (1) 5 min isocratic at 5% B; (2) linear program to 40% B over 90 min and then (3) isocratic at 40% B for 5 min; (4) to 90% B over 5 min; (6) isocratic at 90% B for 5 min; and (6) resetting to 5% B in 20 min. The mass spectrometer was operated in positive-ion nanoelectrospray (nanoESI) mode with source voltage of 2.0 kV and ion-transfer tube temperature of 275 °C. Full-scan mass spectra (MS) were acquired at 60,000 resolution in the Orbitrap and up to 20 MS-dependent tandem mass spectra (MS/MS) were obtained in the ion trap for each full spectrum acquired using collision-induced dissociation (CID) of multiply-charged ions (z ≥ 2). Dynamic exclusion was set for 60 s after an ion was selected for fragmentation. Two technical replicates were run for each sample.

### 2.5. Data Processing and Statistical Analysis

MS/MS spectra were searched against the UniProt protein sequence database (species: *Rattus norvegicus*, 29938 entries) using the Mascot search engine (version 2.6.2; Matrix Science, Boston, MA, USA) run from Proteome Discoverer (version 2.3; Thermo Fisher Scientific). A parent ion mass tolerance and fragment ion mass tolerance were set to 25 ppm and 0.80 Da respectively, and we allowed only one missed cleavage in our search filters. Cysteine carbamidomethylation was indicated as fixed modification and methionine oxidation was designated as variable modification. We used Scaffold software (version 4.9.0, Proteome Software Inc.; Portland, OR, USA) to validate our search results using the Peptide Prophet [24] and Protein Prophet [25] algorithms requiring over 95% and 99% probabilities, respectively, and at least two identified unique peptides for each protein. We have also deposited our data to the ProteomeXchange Consortium [26] by the PRIDE partner repository (assigned dataset identifier: PXD010851). Our label-free quantification relied on spectral counting [23] built into the Scaffold software, and *p* < 0.05 was considered significantly different using unpaired *t*-tests for statistical comparison between sample categories. We also considered twofold change in spectral counts as a threshold of biological effect. Missing values, if any, were handled using Scaffold’s default method and settings. The identified E_2_-regulated proteins were submitted to Ingenuity Pathway Analysis^®^ (IPA^®^, QIAGEN, Redwood City, CA, USA; https://www.qiagenbioinformatics.com/products/ingenuity-pathway-analysis/) to derive bioinformatics annotations along with potential protein interaction networks, as well as associated biological functions and processes. Overlaps of *p*-values were reported from IPA^®^’s calculations using the right-tailed Fisher’s exact test [27].

## 3. Results

From the collected raw data files, protein database search by Mascot within Proteome Discoverer and validation by Scaffold’s algorithms resulted in 1680 actual protein identifications (in 1236 clusters) with 0.01% and 0.4% false discovery at the peptide and protein levels, respectively, according to a decoy-based method of estimation (Table S1). Biological processes and molecular processes associated with the identified rat retina proteins, as well as their cellular localization were summarized in Figure S1. Of the 1680 proteins identified using stringent criteria described in Section 2.5 and using spectral counting as the method of label-free quantification, 158 were up-regulated, while 173 proteins were down-regulated by E_2_ in the retina of OVX rats (Table S2).

**Figure 1:**
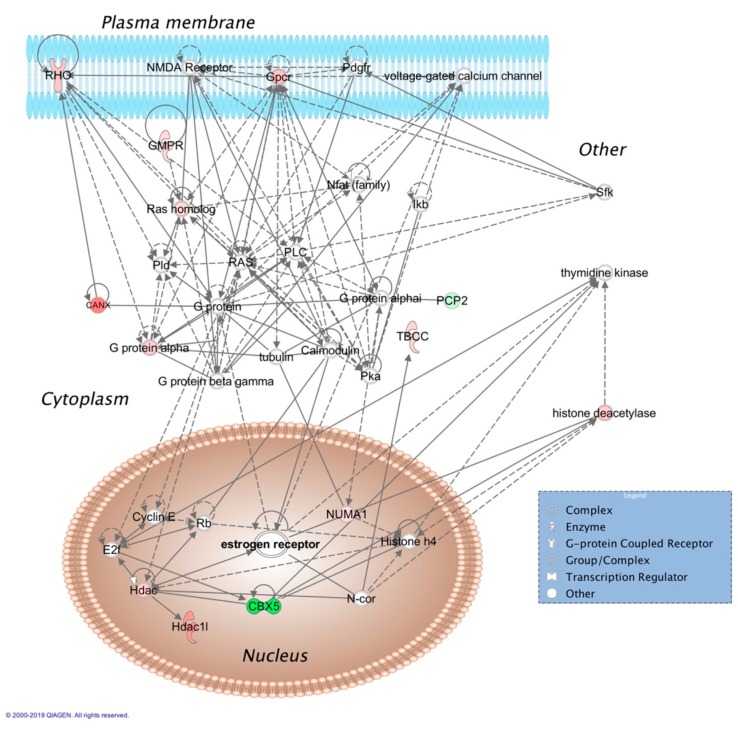
Cell morphology, cell to cell signaling, cellular growth, function, and morphology-related molecular interaction network assembled from retina proteins impacted by topical E_2_ to OVX Brown Norway rats. The shapes (see legend in blue box) represent molecular classes of the regulated proteins. In the network, red and green colors denote upregulation and downregulation in response to E2 treatment, respectively. The intensity of color indicates the relative magnitude of fold change in protein expression pattern. Solid and dashed lines represent direct and indirect interactions, respectively. Abbreviations: CANX, calnexin; CBX5, chromobox 5; E2f, E2F transcription regulator; ERK, extracellular signal-regulated kinase; Gpcr, G-protein coupled receptor; GMPR, guanosine monophosphate reductase; Hdac, histone deacetylase; Ikb, Iκ-B; MAPK, mitogen-activated protein kinase; N-cor, nuclear receptor corepressor; Nfat, nuclear factor of activated T-cells; NMDA, N-methyl-D-aspartic acid; NUMA1, nuclear mitotic apparatus 1; PCP2, Purkinje cell protein 2; Pdgfr, platelet-derived growth factor receptor; Pka, protein kinase A; PLC, phospholipase C; Pld, phospholipase D; Rb, Rb tumor suppressor; RHO, rhodopsin; Sfk, Src family kinase; TBCC, tubulin folding cofactor C.

**Figure 2:**
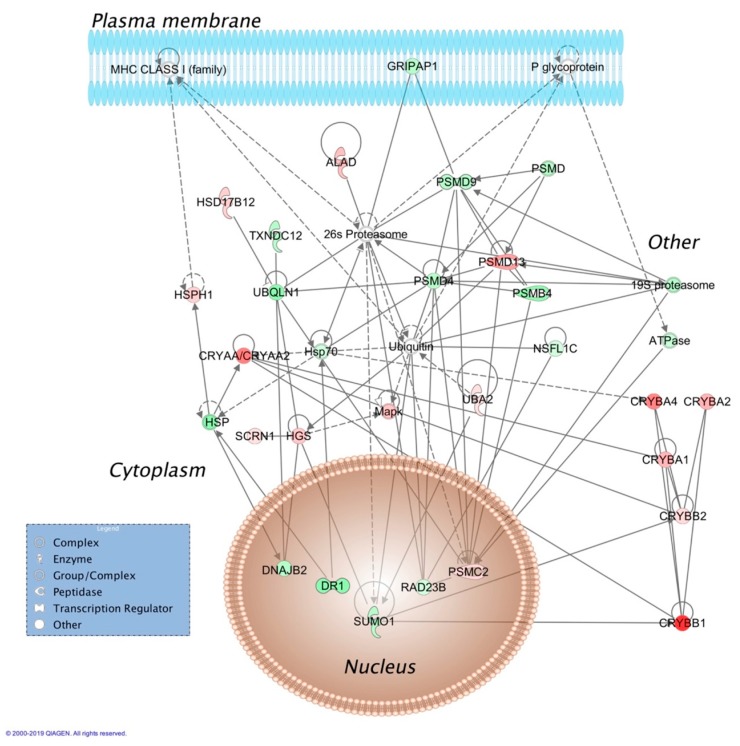
Molecular interaction network associated with development disorder, ophthalmic disease, organismal injury, and abnormalities assembled from retina proteins impacted by E2 given in eye drops to OVX rats. See explanation of shapes, colors and lines in the legend of Figure 1. Abbreviations: ALAD, aminolevulinate dehydratase; ATPase, adenosine triphosphatase; CRYAA/CRYAA2, α-crystallin A/A2; CRYBA1, CRYBA2, CRYBA4, CRYBB1, and CRYBB2, β-crystallin A1, A2, A4, B1 and B2; DNAJ32, DnaJ heat shock protein family (Hsp40) member B2; DR1, down regulator of transcription 1; GRIPA1, glutamate receptor-interacting protein associated protein 1; HGS, hepatocyte growth factor-regulated tyrosine kinase substrate; HSD17B12, hydroxysteroid 17-β dehydrogenase 12; HSP, heat shock protein; Hsp70, heat shock protein 70; HSPH1, heat shock protein family H (Hsp110) member 1; NSFL1C, N-ethylmaleimide-sensitive factor L1 cofactor; PSMB4, proteasome subunit-β 4; PSMC2, proteasome 26S subunit, non-ATPase 2; PSMD, proteasome 26S subunit, non-ATPase; PSMD4, PSMD9 and PSMD13, proteasome 26S subunit, non-ATPase 4, 9 and 13; RAD23B, nucleotide excision protein RAD23 homolog B; SCRN1, secernin 1; SUMO1, small ubiquitin-like modifier 1; TXNDC12, thioredoxin domain containing 12; UBA2, ubiquitin-like modifier activating enzyme 2; UBQLN1, ubiquilin 1.

**Figure 3:**
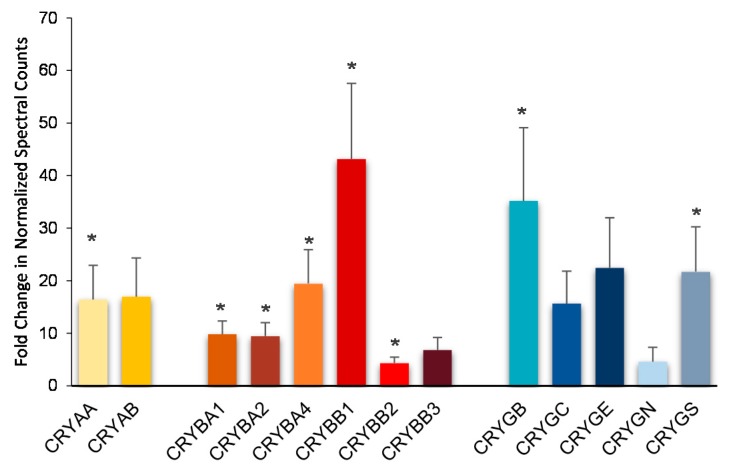
Upregulation of all crystallins was detected using normalized MS/MS spectral counts (SC) as quantitative measures in the retina of OVX rats after treatment with E2 given in eye drops. Fold changes are displayed as averages ± standard error (*n* = 5/group). Asterisks (*) indicate statistically significant differences of SC between treatment as control using unpaired *t*-tests.

Top molecular and cellular functions, as well as physiological development and function influenced by E_2_ based on bioinformatics analyses (IPA^®^) were summarized in Table 1. Two representatives of the 18 protein interaction networks assembled using E_2_-regulated retinal proteins as inputs for IPA^®^ are displayed in Figure 1 and Figure 2, and selected canonical pathways and targets associated with these networks are listed in Table 2 and Table 3, respectively. Details on the rest of the networks are included in the supplementary material (Figures S2–S18). Networks shown in Figure 2 called our attention to crystallins as potential major players orchestrating the hormone’s action strongly related to development disorder, ophthalmic disease, as well as organismal injury and abnormalities. Indeed, we found that all covered isoforms of these proteins were upregulated in the retina upon topical E_2_ treatments, as shown in Figure 3.

## 4. Discussion

Previous physiological and pharmacological research have highlighted estrogen as a key regulator of retinal health [17,18] and we have shown beneficial broad-spectrum neuroprotective effects of the topically applied E_2_ in OVX rats that have no appreciable level of circulating hormone [20]. For the proteomics study presented here, we obtained samples relying on the previously employed treatment protocol. This will afford correlation of the results with those of apoptotic cell death in the ganglion cell layer and behavioral testing of visual performance published earlier [20]. Our results have enabled the assembly of protein networks that captured systems-level impacts of the hormone on the retina for the first time. It is expected that the wealth of information from this report will be valuable for neuroprotective ophthalmic therapy development relying on repurposing [6,8], polypharmacology [9], and target-focused new drug discovery [28], as well as for actual preventative medication aimed specifically at preserving the function of retinal ganglion cells (RGCs) from the postmenopausal impact of estrogen deficiency [29] alike. This expectation is justified especially in the context of emerging results from similar omics-driven profiling experiments on neurodegenerative diseases of the human eye [30] or animal models thereof [31,32,33,34].

We point out that our experimental approach focused on the identification of E2-regulated proteins was based on detecting changes in normalized spectral counts and not based on fold changes in absolute protein levels [20,23]. For proteins solubilized from a complex cellular proteome by an 8 M aqueous urea solution, linear relationships of normalized spectral counts and protein levels have been shown comprehensively over a wide dynamic range [35]. Therefore (albeit with some degree of caution about some specific proteins as noted in the heading of Table S2), ≥2-fold changes in normalized spectral counts [36] should have enabled reliable detections of statistically significant overall trends in up- and downregulation for the urea-solubilized retina proteins we report here.

A mechanistically meaningful target engagement by the E_2_ delivered to the retina was revealed by the network shown in Figure 1, which implicated activation of signaling orchestrated by nuclear estrogen receptor (ER) as its top canonical pathway in association with cell morphology, cell to cell signaling, cellular growth, function and morphology as underlying biology. Messenger ribonucleic acid (mRNA) for ER expression has been shown in the eye [37], and the protein was detected in the retina [38,39]. Stimulation of extracellular signal-regulated kinase/mitogen-activated protein kinase (ERK/MAPK) signaling (listed in the inset table of the figure), a well-known mechanism critical for estrogen neuroprotection [40], was an additional pathway strongly linked to this network. Therefore, this previously shown ER-mediated neuroprotective effect of E2 in the retina and RGCs through ERKs [41,42] was supported by our data.

Another network shown in Figure 2, linked to development disorder, ophthalmic disease, organismal injury, and abnormalities by IPA^®^, apparently captured the profound impact of E_2_ on proteasome activity implicating the hormone’s role in the clearance of damaged proteins [43]. Moreover, there were several crystallin isoforms of α-crystallin (CRYAA/CRYAA2) and β-crystallin (CRYBA1, CRYBA2, CRYBA4, CRYBB1, and CRYBB2) strongly associated with this network. In other networks displayed in Figures S4 and S14 specifically linked to ophthalmic disease, γ-crystallin B (CRYGB) and γ-crystallin S (CRYGS) were also present. Crystallins represent a rather heterogeneous group of proteins with diverse functions in the eye [44,45]. In the retina, changes in their expression profile are considered an indication of a significant role of these proteins to maintain homeostasis [46]. Our mass spectrometry-based proteomics study has shown that all covered isoforms of crystallins are upregulated in the retina by E_2_ administered in eye drops, as summarized in Figure 3.

The α-crystallins (CRYAA and CRYAB) interact with many proteins including other crystallins, cytoskeletal proteins, and proteins involved in inflammatory, signaling, angiogenic, and apoptotic pathways [47]. CRYAA and CRYAB function as molecular chaperones that prevent aberrant protein interactions [48,49]. Therefore, they protect key proteins and stabilize cells in the retina, as well as inhibit apoptosis-induced cell death [44,46]. Their overexpression promotes survival of RGCs injured by ocular hypertension and optic nerve crush [50,51]. On the other hand, a decrease of CRYAA has been associated with retinal dystrophy [52] and glaucomatous optic neuropathy [44]. However, intravitreal injection of CRYAB at the time of the intraocular pressure (IOP) increase rescued RGCs in a rat model of glaucoma [53]. The latter study also provided proteomic evidence that CRYAB injection upregulated all (α, β and γ) subclasses of crystallins in the retina, which induced the broad neuroprotective effects observed. Using the cauterization of episcleral veins to achieve chronic IOP elevation in rats as an experimental glaucoma model, a recent mass spectrometry-based proteomics experiment has established a potential correlation of age-related glaucomatous damage and the absence of all isoforms of the crystallin protein in the retina [54].

Compared to α-crystallins, much less has been understood about the retinal function of β- and γ-cystallins [43,44,55]. Like CRYAA and CRYBB, β-crystallin genes are downregulated at both transcriptional and protein levels in rat retinas with ocular hypertension [56], and upregulation of β-crystallins has been linked to retina neuroprotection and axonal regeneration [55]. Moreover, CRYBB2 upregulation during retinal regeneration in vitro and its localization in RGCs and their axons (including growth cones and filopodia) have also been reported [57]. Perhaps the most compelling evidence of the significance of CRYBB2 for retina neuroprotection has been that its intravitreal injection at the time of IOP elevation improves retinal ganglion cell survival in a rat model of glaucoma combined with mass spectrometry-based proteomics experiments [58]. Overall, the latter revealed CRYBB2′s impact on calcium-dependent cell signaling pathways with profound effect on apoptosis and gene regulation, in which annexin A5, Ca^2+^-transporting ATPase 1, and various histone proteins supposedly play a major role. From our proteomics results, we could unequivocally confirm the connection of CRYBB2 upregulation in the rat retina by E_2_ with that of plasma membrane Ca^2+^-transporting ATPase 1 (AT2B1, Table S2), which may be associated with an estrogenic neuroprotective effect elicited by a non-genomic mechanism [16,59]. Beyond defense against glaucomatous neurodegeneration by β-cystallin(s), their protective role in type 2 epithelial-to-mesenchymal transition of retinal-pigment epithelial cells occurring in dry age-related macular degeneration has been shown recently [60]. However, all crystallins were found to be upregulated in a neonatal mouse model of oxygen-induced retinopathy [61,62,63,64], which indicated a developmental stage-dependent control of their expression. Nevertheless, our data summarized in Figure 3 support the observation about a highly correlated group of α-, β-, and γ-crystallin genes [65]. Therefore, it is not surprising that crystallin-based cell survival strategies have been proposed to protect and rescue RGCs from degeneration associated with glaucomatous and other optic neuropathies [50]. However, therapeutic protein delivery to the retina faces many obstacles [66,67,68] compared to small molecule therapy such as E_2_ delivered topically [20], which would induce the desired changes in protein expression.

Many potential associations and pathways captured by Figure 1 and Figure 2, as well as by the additional protein interaction networks of Figures S2–S17 have not been explored by research specifically addressing the retinal milieu. However, we anticipate that systems insights made possible by our results will guide future hypothesis-driven experiments focusing on the “estrogenic retina” [17,18], including potential therapeutic application of estrogens as topically delivered broad-spectrum retina neuroprotectants [20,69].

In conclusion, our mass spectrometry-based proteomics study has revealed a new, extended set of retina proteins significantly affected by the treatment of OVX Brown Norway rats with E_2_ administered in eye drops. Data provided evidence not only on multiple target engagements, but also afforded detailed bioinformatics analyses focusing on protein interaction networks and biological processes they represented. Strong associations of the impact with nervous system development and function, tissue development, organ development, as well as visual system development and function, argue for further consideration of estrogens for neuroprotective ophthalmic pharmacotherapy targeting the retina.

## Figures and Tables

**Table 1:** (**A**) Molecular and cellular functions, as well as (**B**) physiological system development and function represented by significantly affected retina proteins identified by label-free quantitative proteomics in ovariectomized (OVX) Brown Norway rats that received topical E_2_ in eye drops.

(**A**)		
**Represented Process**	**Number of Associated Molecules**	***p*-Value of Overlap**	
Cellular function and maintenance	123	4.62·10^−3^–5.05·10^−13^
Cellular assembly and organization	117	4.79·10^−3^–5.05·10^−13^
DNA replication, recombination and repair	21	3.82·10^−3^–1.36·10^−11^
Small-molecule biochemistry	82	4.83·10^−3^–1.36·10^−11^
Nucleic acid metabolism	37	4.83·10^−3^–1.36·10^−11^
(**B**)		
**Associated Physiology**	**Number of Linked Molecules**	***p*-Value of Overlap**
Nervous system development and function	92	4.62·10^−3^–5.56·10^−8^
Tissue development	74	4.24·10^−3^–3.40·10^−6^
Organismal development	60	4.24·10^−3^–3.40·10^−6^
Organ development	29	4.24·10^−3^–1.96·10^−4^
Visual system development and function	18	4.24·10^−3^–1.96·10^−4^

**Table 2:** Canonical pathways and targets associated with the network shown in Figure 1.

Canonical Pathway	Targets	Z-Score ^1^	*p*-Value
Estrogen receptor signaling	estrogen receptor; G protein; Hdac; histone deacetylase; N-cor; Pka; PLC; Ras; Ras homolog; Sfk	N/A	6.03·10^−3^
ERK/MAPK signaling	estrogen receptor; G protein; Nfat; Pka; PLC; Ras; Ras homolog; Sfk	1.8	2.40·10^−2^

^1^ Positive value: Activation of the canonical pathway; N/A: No prediction can be made.

**Table 3:** Canonical pathways and targets associated with the network shown in Figure 2.

Canonical Pathway	Targets	Z-Score ^1^	*p*-Value
Protein ubiquitination	19S proteasome; 26S proteasome; ATPase; CRYAA/CRYAA2; DNAJB2; Hsp70; HSP; HSPH1; MHC CLASS I (family); PSMB4; PSMC2; PSMD4; PSMD9; PSMD13; PSMD; Ubiquitin	N/A	2.86·10^−6^
Clathrin-mediated endocytosis	ATPase; HSP; HGS; HSP70; Ubiquitin	N/A	4.14·10^−9^
Synaptogenesis signaling	ATPase; HSP70; HSP; MAPK	1.1	2.20·10^−4^

^1^ Positive value: Activation of the canonical pathway; N/A: No prediction can be made.

## Supplementary Materials

The following are available online at https://www.mdpi.com/1999-4923/12/2/101/s1, Table S1: List of identified and validated proteins (Scaffold 4.9.0: >95% and >99% probability by Peptide Prophet and protein Prophet, respectively, with minimum of two unique peptides) in the retina, along with their gene ontology (GO) annotations, Figure S1: Biological processes and molecular processes associated with the identified rat retina proteins, and cellular localization of the identified rat retina proteins, Table S2: A list of E2-regulated retinal proteins in OVX rats, Figure S2: IPA^®^ network linked to molecular transport, RNA posttranscriptional modification, RNA trafficking, Figure S3: IPA^®^ network linked infectious disease, organismal injury and abnormalities, RNA posttranslational modification, Figure S4: IPA^®^ network linked to cancer, infectious disease, organismal injury and abnormalities, Figure S5: IPA^®^ network linked to cellular assembly and organization, cellular function and maintenance, connective tissue disorder, Figure S6: IPA^®^ network linked to cell to cell signaling and interaction, cellular function and maintenance, nervous system development and function, Figure S7: IPA^®^ network linked to carbohydrate metabolism, cellular compromise, cellular function and maintenance, Figure S8: IPA^®^ network linked to cellular assembly and organization, energy production, nucleic acid metabolism, Figure S9: IPA^®^ network linked to cell morphology, cellular assembly and organization, nervous system development and function, Figure S10: IPA^®^ network linked to developmental disorder, hereditary disorder, metabolic function, Figure S11: IPA^®^ network linked to gene expression, RNA damage and repair, RNA post translational modification and repair, Figure S12: IPA^®^ network linked to nucleic acid metabolism, protein synthesis, small-molecule biochemistry, Figure S13: IPA^®^ network linked to cellular assembly and organization, cellular function and maintenance, and tissue development, Figure S14: IPA^®^ network linked to cell morphology, cellular function and maintenance, and cellular compromise, Figure S15: IPA^®^ network linked to cancer, cellular growth and proliferation, organismal injury and compromise, Figure S16: IPA^®^ network linked to amino acid metabolism, cellular assembly and organization, small-molecule biochemistry, Figure S17: IPA^®^ network linked to cell death and survival, cellular development, cellular growth and proliferation, Figure S18: Overlapping IPA^®^ networks.
