# Supplementary material for: 17β-Estradiol Delivered in Eye Drops: Evidence of Impact on Protein Networks and Associated Biological Processes in the Rat Retina through Quantitative Proteomics
Source: Pharmaceutics. 2020 Jan 27;12(2):101. doi: 10.3390/pharmaceutics12020101 (PMC7076522; doi:10.3390/pharmaceutics12020101)
Supplement: Supplementary file 1 [file pharmaceutics-12-00101-s001.zip › pharmaceutics-685990_SupportingMaterialFiguresS1-S18.pdf]

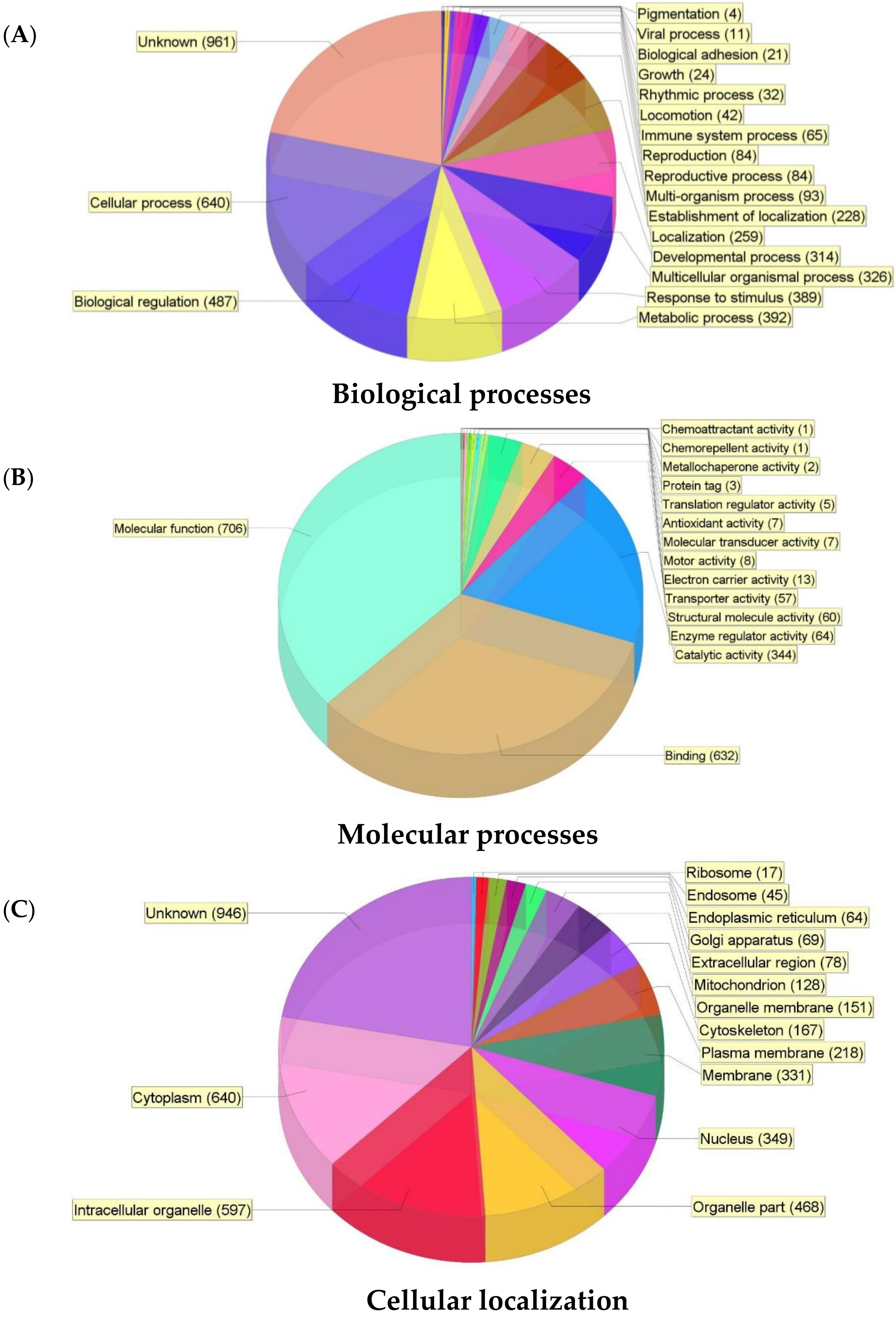

Figure S1. Biological processes and molecular processes associated with the identified rat retina proteins, and cellular localization of the identified rat retina proteins. (**A**) Biological processes; (**B**) Molecular processes; (**C**) Cellular localization. Information was compiled from validated identifications using Scaffold (version 4.9.0; Proteome Software, Portland, OR, USA).

(**A**)

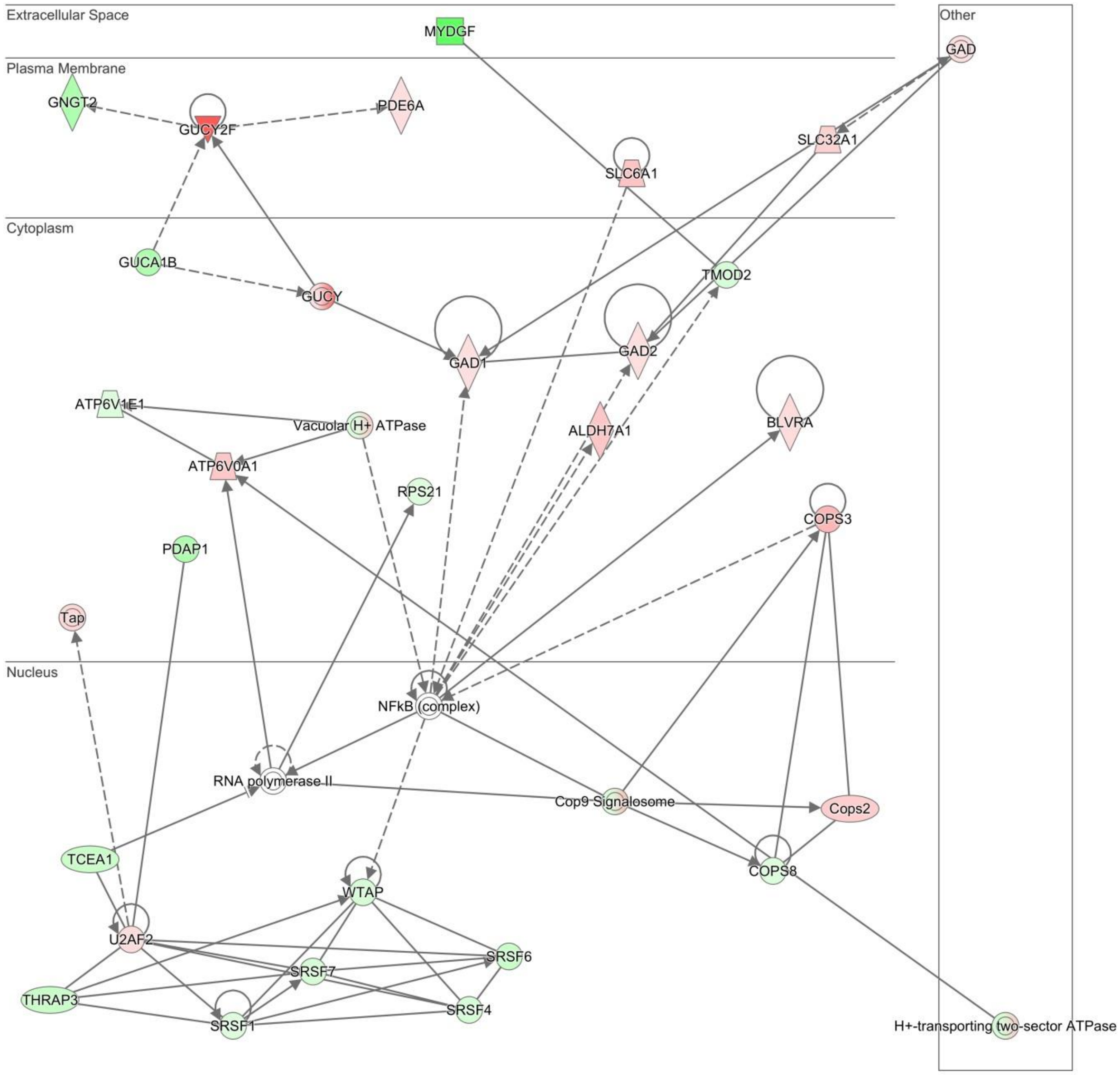

(**B**)

**Canonical pathway**	**Targets**	**Z-score**	***p*-value**
Sirtuin signaling pathway	H^+-transporting two-sector ATPase, NFkB (complex), RNA polymerase II	-0.3	3.26E-07
GABA receptor signaling	GAD1, AD2, GAD, GUCY, GUCY2F, SLC32A1, SLC6A1	N/A	1.04E-05
Protein kinase A signaling	NFkB (complex), PDE6A	2.3	6.03E-03

Figure S2. IPA® network linked to molecular transport, RNA posttranscriptional modification, RNA trafficking (**A**). The network was assembled from E_2-regulated proteins in the retina of OVX Brown Norway rats through identification by label-free shotgun proteomics. In the network, red and green colors denote upregulation and downregulation in response to E2 treatment, respectively. The intensity of color indicates the relative magnitude of fold change in the protein's expression pattern. Solid and dashed lines represent direct and indirect interactions, respectively. For further details on symbols and relationships, see http://qiagen.force.com/KnowledgeBase/articles/Basic_Technical_Q_A/Legend. Inset table (**B**): Top canonical pathways and their corresponding molecular targets associated with the network shown. Z-scores indicate activation or suppression of the corresponding pathway (positive or negative values, respectively; N/A: no prediction can be made).

(**A**)

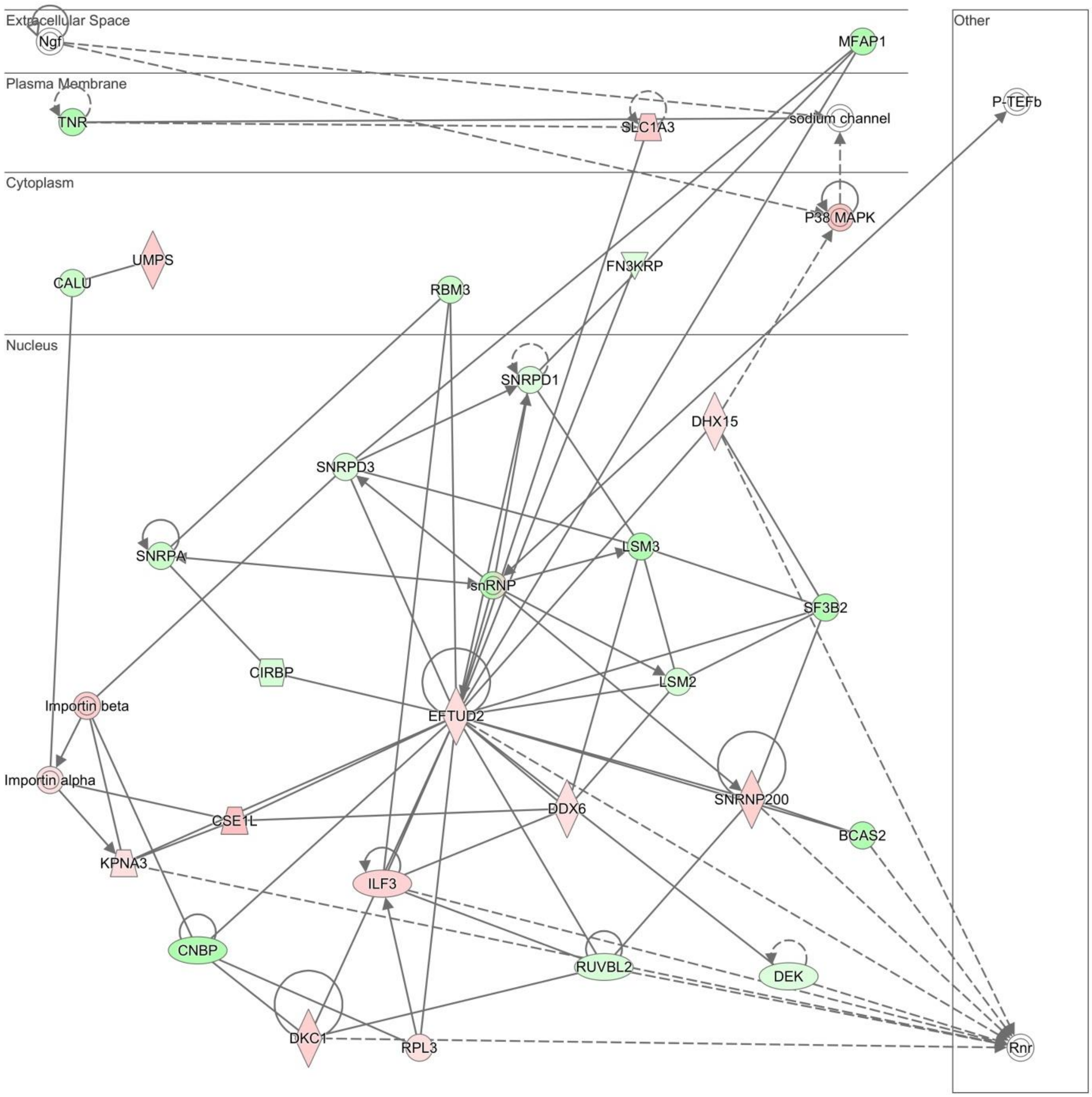

(**B**)

**Canonical pathway**	**Targets**	**Z-score**	***p*-value**
EIF2 signaling	P38 MAPK, Rnr, RPL3	1.3	1.78E-02
Axonal guidance signaling	Ngf, P38 MAPK	N/A	1.76E-01
RAN signaling	CSE1L, Importin alpha, Importin beta, KPNA3	N/A	9.08E-05

Figure S3. IPA® network linked to infectious disease, organismal injury and abnormalities, RNA post- transcriptional modification (**A**). The network was assembled from E_2-regulated proteins in the retina of OVX rats through identification by label-free shotgun proteomics. In the network, red and green colors denote upregulation and downregulation in response to E2 treatment, respectively. The intensity of color indicates the relative magnitude of fold change in the protein's expression pattern. Solid and dashed lines represent direct and indirect interactions, respectively. For further details on symbols and relationships, see http://qiagen.force.com/KnowledgeBase/articles/Basic_Technical_Q_A/Legend. Inset table (**B**): Top canonical pathways and their corresponding molecular targets associated with the network shown. Z-scores indicate activation or suppression of the corresponding pathway (positive or negative values, respectively; N/A: no prediction can be made).

(A)

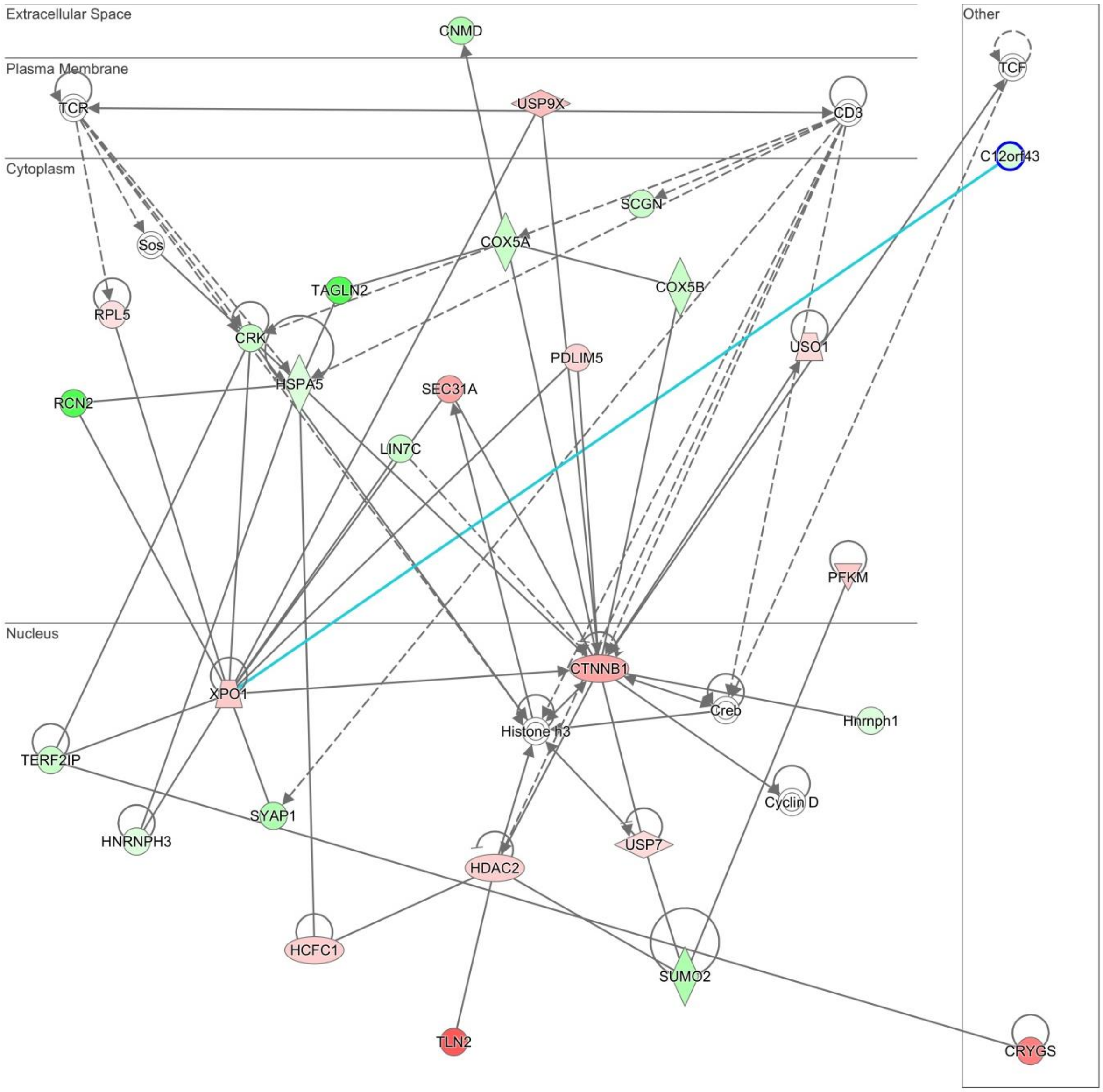

(B)

Canonical pathway	Targets	Z-score	*p*-value
Glucocorticoid receptor signaling	CD3, Creb, Histone h3, HSPA5, Sos, TCR	N/A	
Synaptogenesis pathway	Creb, CRK,CTNNB1, Sos	1.1	2.20E-04
ERK/MAPK signaling	Creb, CRK, Histone h3, Sos, TLN2	1.8	2.40E-02

Figure S4. IPA® network linked to cancer, infectious disease, organismal injury and abnormalities (**A**). The network was assembled from E_2-regulated proteins in the retina of OVX rats through identification by label-free shotgun proteomics. In the network, red and green colors denote upregulation and downregulation in response to E2 treatment, respectively. The intensity of color indicates the relative magnitude of fold change in the protein's expression pattern. Solid and dashed lines represent direct and indirect interactions, respectively. For further details on symbols and relationships, see http://qiagen.force.com/KnowledgeBase/articles/Basic_Technical_Q_A/Legend. Inset table (**B**): Top canonical pathways and their corresponding molecular targets associated with the network shown. Z-scores indicate activation or suppression of the corresponding pathway (positive or negative values, respectively; N/A: no prediction can be made).

(A)

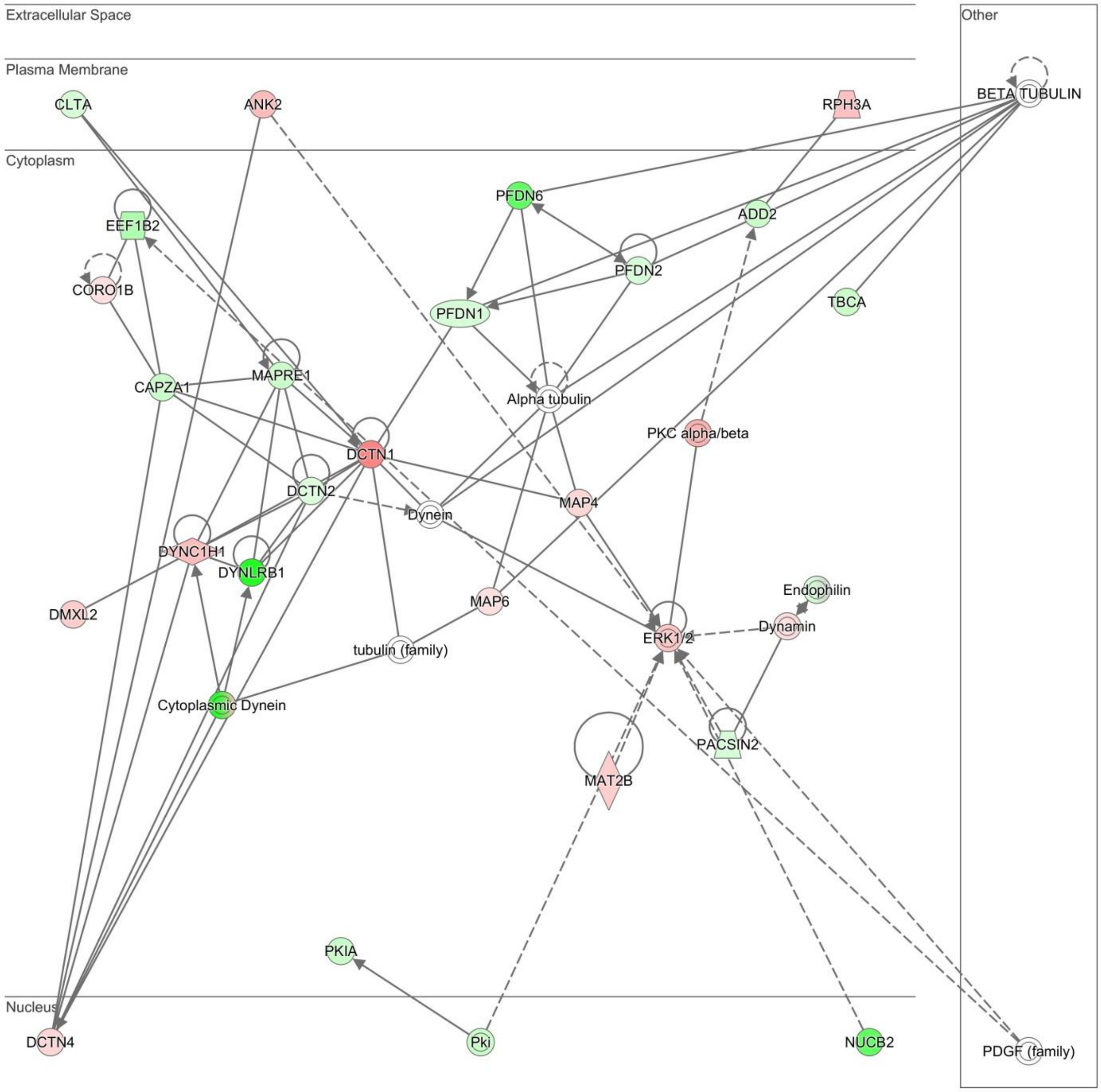

(B)

Canonical pathway	Targets	Z-score	*p*-value
Clathrin-mediated endocytosis	CLTA, Dynamin, Endophilin, PDGF (family)	N/A	4.14E-09
Sirtuin signaling pathway	Alpha tubulin, ERK1/2, tubulin (family)	-0.3	3.26E-07

Figure S5. IPA® network linked to cellular assembly and organization, cellular function and maintenance, connective tissue disorder (**A**). The network was assembled from E_2-regulated proteins in the retina of OVX rats through identification by label-free shotgun proteomics. In the network, red and green colors denote upregulation and downregulation in response to E2 treatment, respectively. The intensity of color indicates the relative magnitude of fold change in the protein's expression pattern. Solid and dashed lines represent direct and indirect interactions, respectively. For further details on symbols and relationships, see http://qiagen.force.com/KnowledgeBase/articles/Basic_Technical_Q_A/Legend. Inset table (**B**): Top canonical pathways and their corresponding molecular targets associated with the network shown. Z-scores indicate activation or suppression of the corresponding pathway (positive or negative values, respectively; N/A: no prediction can be made).

(**A**)

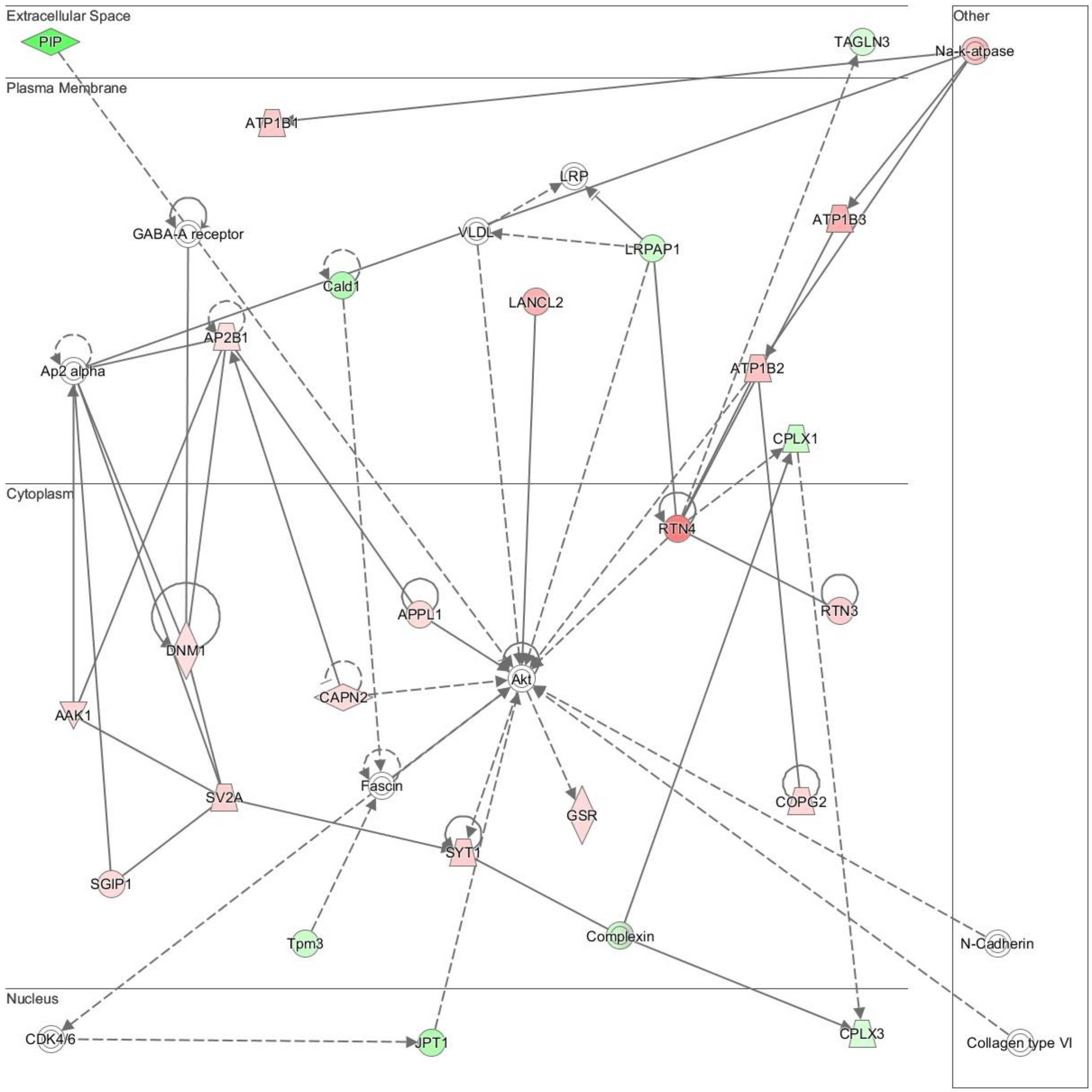

(**B**)

**Canonical pathway**	**Targets**	**Z-score**	***p*-value**
Clathrin-mediated endocytosis	AAK1, Ap2 alpha, AP2B1, DNM1	N/A	4.14E-09
Synaptogenesis pathway	Akt, Ap2 alpha, AP2B1, COMPLEXIN, CPLX1, CPLX3, LRP, N-Cadherin, SYT1	1.1	2.20E-04
Wnt/Beta catenin signaling	Akt, APPL1, LRP, N-cadherin		

Figure S6. IPA® network linked to cell to cell signaling and interaction, cellular function and maintenance, nervous system development and function (**A**). The network was assembled from E_2-regulated proteins in the retina of OVX rats through identification by label-free shotgun proteomics. In the network, red and green colors denote upregulation and downregulation in response to E2 treatment, respectively. The intensity of color indicates the relative magnitude of fold change in the protein's expression pattern. Solid and dashed lines represent direct and indirect interactions, respectively. For further details on symbols and relationships, see http://qiagen.force.com/KnowledgeBase/articles/Basic_Technical_Q_A/Legend. Inset table (**B**): Top canonical pathways and their corresponding molecular targets associated with the network shown. Z-scores indicate activation or suppression of the corresponding pathway (positive or negative values, respectively; N/A: no prediction can be made).

(A)

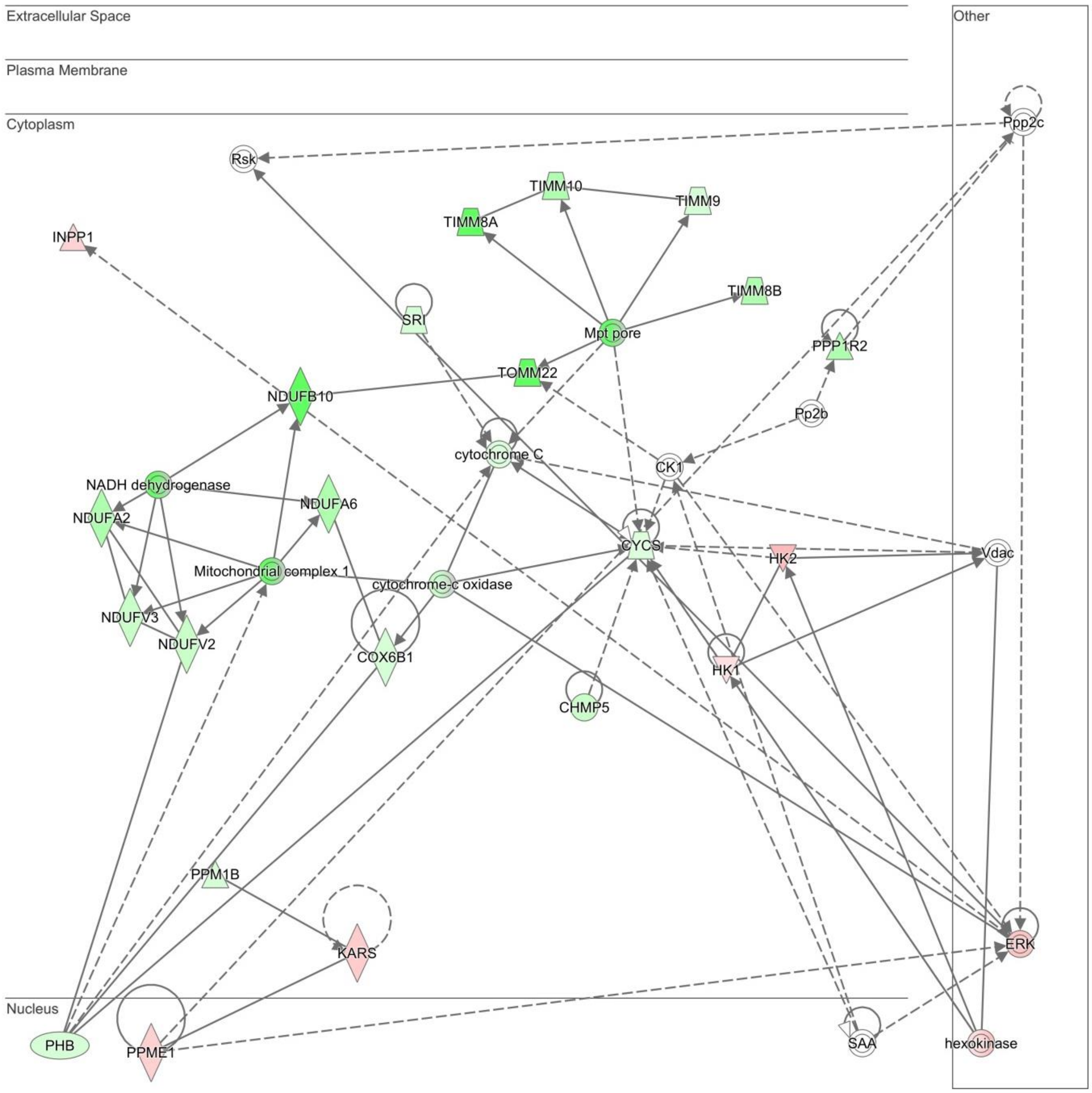

(B)

Canonical pathway	Targets	Z-score	*p*-value
Sirtuin signaling pathway	cytochrome C, ERK, Mitochondrial complex 1, Mpt pore, NADH dehydrogenase, NDUFA2, NDUFA6, NDUFB10, NDUFV2, NDUFV3, TIMM9, TIMM10, TIMM8A, TIMM8B, TOMM22, Vdac	-0.3	3.26E-07
Oxidative phosphorylation	COX6B1, CYCS, cytochrome C, cytochrome-c oxidase, Mitochondrial complex1, NADH dehydrogenase, NDUFA2, NDUFA6, NDUFB10, NDUFV2, NDUFV3	-3.6	7.03E-09
ERK/MAPK signaling	ERK, Ppp2c, Rsk	1.8	2.40E-02

Figure S7. IPA® network linked to carbohydrate metabolism, cellular compromise, cellular function and maintenance (**A**). The network was assembled from E_2-regulated proteins in the retina of OVX rats through identification by label-free shotgun proteomics. In the network, red and green colors denote upregulation and downregulation in response to E2 treatment, respectively. The intensity of color indicates the relative magnitude of fold change in the protein's expression pattern. Solid and dashed lines represent direct and indirect interactions, respectively. For further details on symbols and relationships, see http://qiagen.force.com/KnowledgeBase/articles/Basic_Technical_Q_A/Legend. Inset table (**B**): Top canonical pathways and their corresponding molecular targets associated with the network shown. Z-scores indicate activation or suppression of the corresponding pathway (positive or negative values, respectively).

(A)

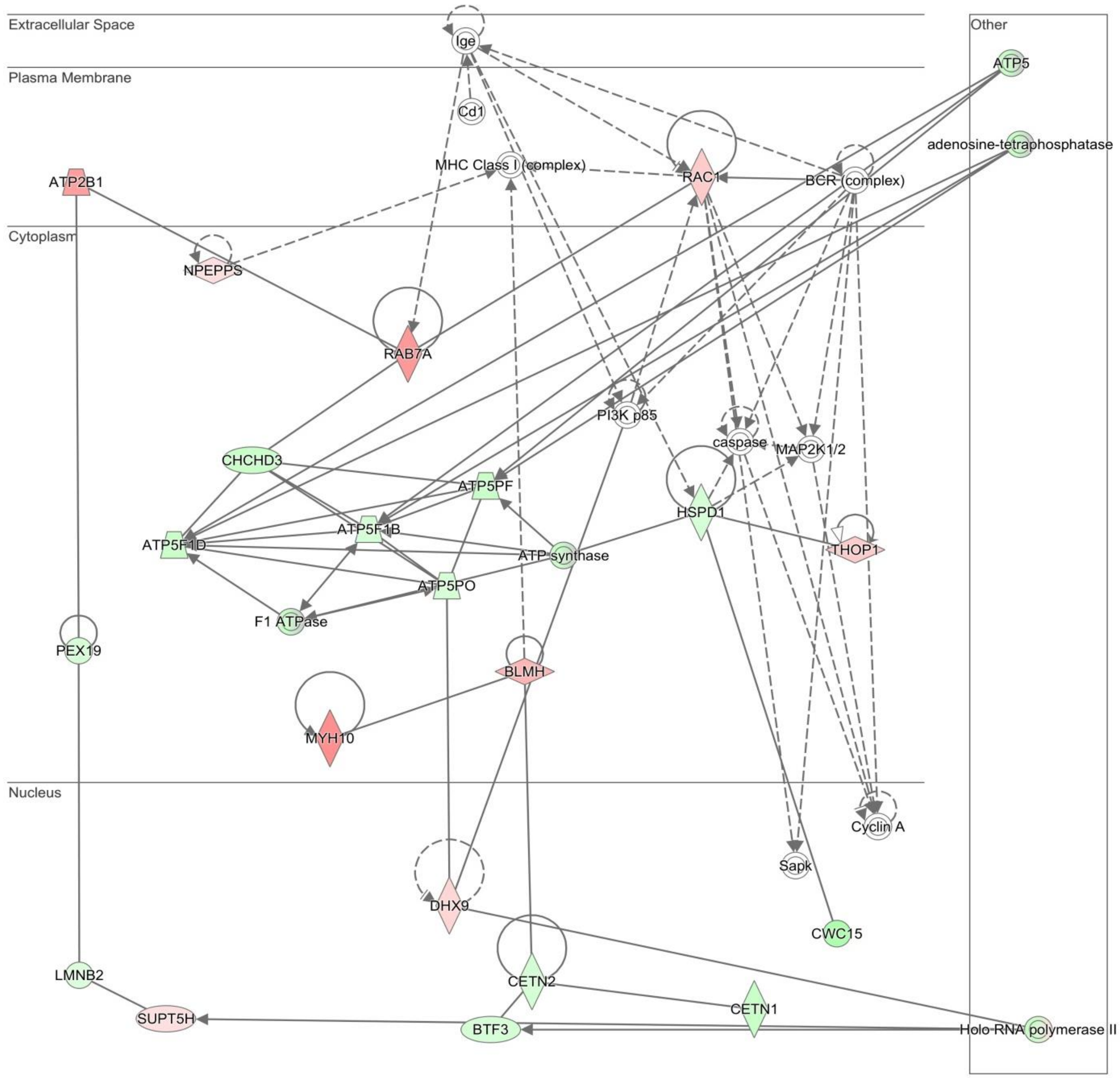

(B)

Canonical pathway	Targets	Z-score	*p*-value
Sirtuin signaling pathway	Adenosine-tetraphosphatase, ATP5, ATP synthase, ATP5F1B, ATP5F1D, ATP5PF, F1 ATPase, Holo RNA pol2, Sapk	-0.3	3.26E-07
Oxidative phosphorylation	Adenosine-tetraphosphatase, ATP5, ATP synthase, ATP5F1B, ATP5F1D, ATP5PF, ATP5PO, F1 ATPase	-3.6	7.03E-09
Mitochondrial dysfunction	Adenosine-tetraphosphatase, ATP5, ATP synthase, ATP5F1B, ATP5F1D, ATP5PF, F1 ATPase, caspase, Sapk	N/A	2.29E-07

Figure S8. IPA® network linked to cellular assembly and organization, energy production, nucleic acid metabolism (**A**). The network was assembled from E_2-regulated proteins in the retina of OVX rats through identification by label-free shotgun proteomics. In the network, red and green colors denote upregulation and downregulation in response to E2 treatment, respectively. The intensity of color indicates the relative magnitude of fold change in the protein's expression pattern. Solid and dashed lines represent direct and indirect interactions, respectively. For further details on symbols and relationships, see http://qiagen.force.com/KnowledgeBase/articles/Basic_Technical_Q_A/Legend. Inset table (**B**): Top canonical pathways and their corresponding molecular targets associated with the network shown. Z-scores indicate activation or suppression of the corresponding pathway (positive or negative values, respectively).

(**A**)

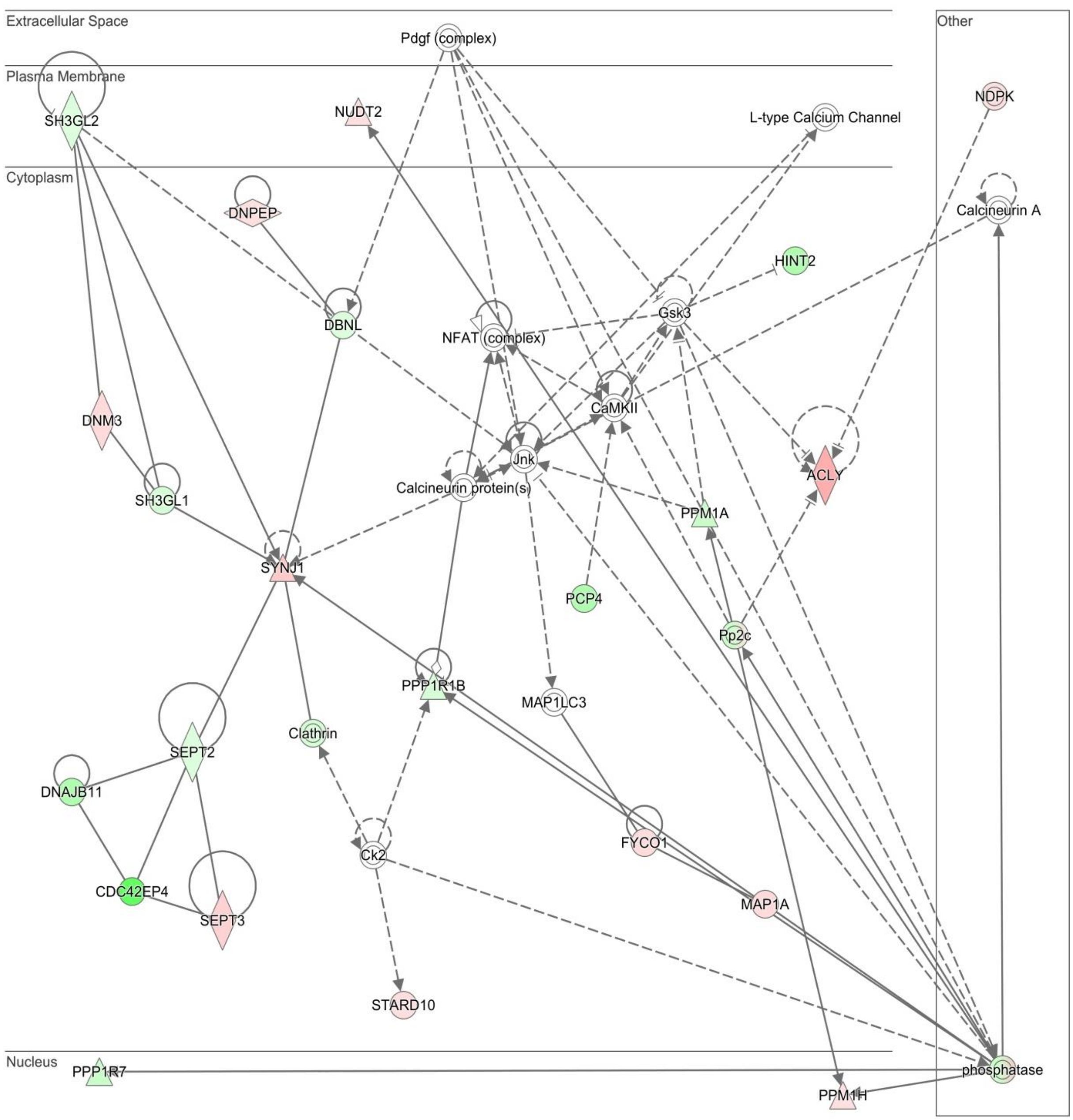

(**B**)

Canonical pathway	Targets	Z-score	*p*-value
Clathrin-mediated endocytosis	Calcineurin A, Calcineurin proteins, CK2, Clathrin, DNM3, Pdgf, phosphatase, SH3GL1, SH3GL2, SYNJ1	N/A	4.14E-09
Protein kinase A signaling	Calcineurin A, Calcineurin proteins, CAMKII, Gsk3, NFAT, phosphatase, PPP1R7, PPP1R1B	2.3	6.03E-03
AMPK pathway	Jnk, NDPK, phosphatase, Pp2c, PPM1A, PPM1H	1.9	3.09E-04

Figure S9. IPA® network linked to cell morphology, cellular assembly and organization, nervous system development and function (**A**). The network was assembled from E_2-regulated proteins in the retina of OVX rats through identification by label-free shotgun proteomics. In the network, red and green colors denote upregulation and downregulation in response to E2 treatment, respectively. The intensity of color indicates the relative magnitude of fold change in the protein's expression pattern. Solid and dashed lines represent direct and indirect interactions, respectively. For further details on symbols and relationships, see http://qiagen.force.com/KnowledgeBase/articles/Basic_Technical_Q_A/Legend. Inset table (**B**): Top canonical pathways and their corresponding molecular targets associated with the network shown. Z-scores indicate activation or suppression of the corresponding pathway (positive or negative values, respectively; N/A: no prediction can be made).

(**A**)

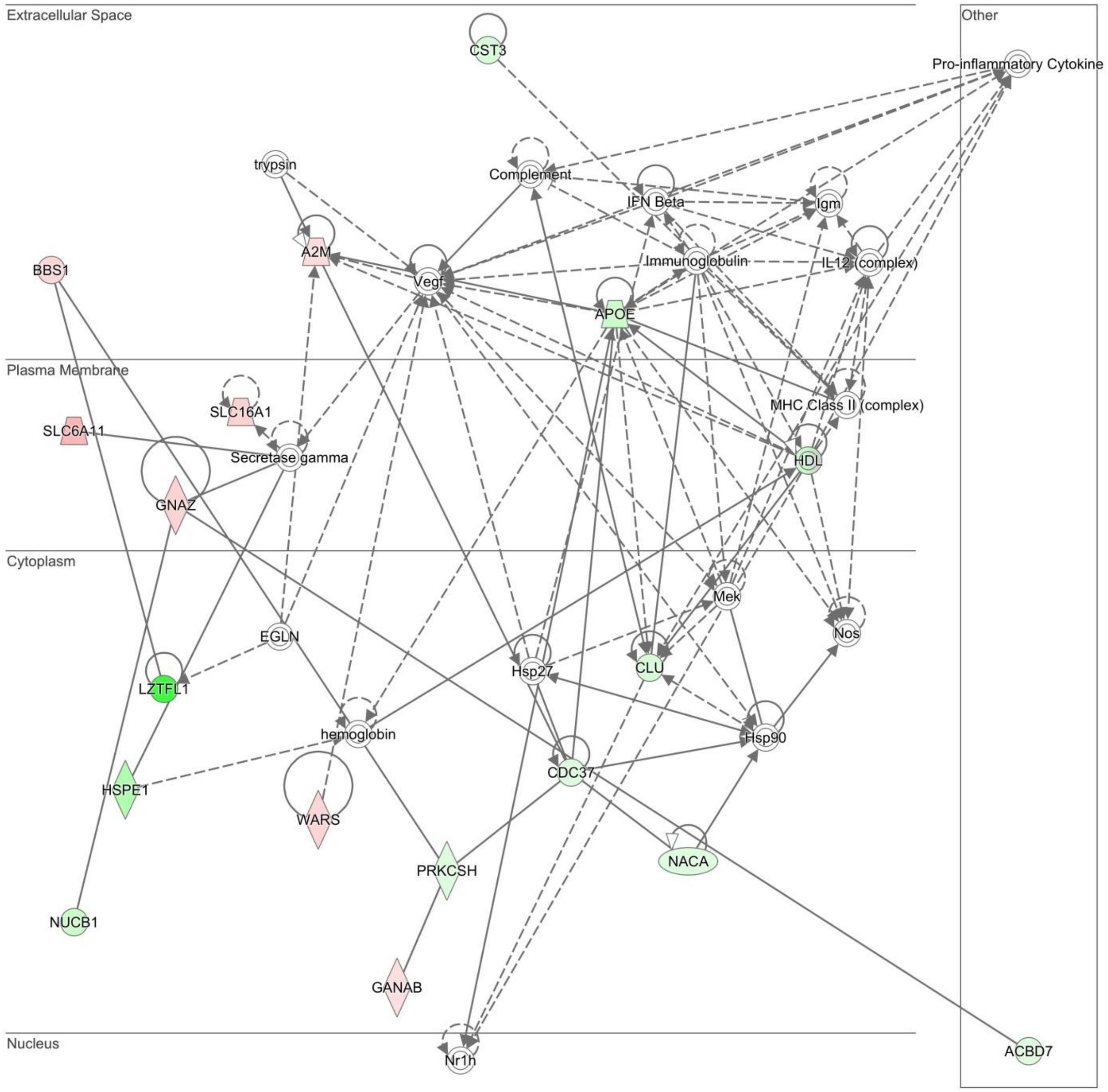

(**B**)

Canonical pathway	Targets	Z-score	*p*-value
Clathrin-mediated endocytosis	APOE, CLU, HDL, Vegf	N/A	4.14E-09
Protein ubiquitination pathway	Hsp27, Hsp90. HSPE1, MHC class II, Pro-inflammatory cytokine	N/A	2.86E-06
Sirtuin signaling pathway	Nos, Nr1h, Pro-inflammatory cytokine	-0.3	3.26E-07

Figure S10. IPA® network linked to developmental disorder, hereditary disorder, metabolic disease (**A**). The network was assembled from E_2-regulated proteins in the retina of OVX rats through identification by label-free shotgun proteomics. In the network, red and green colors denote upregulation and downregulation in response to E2 treatment, respectively. The intensity of color indicates the relative magnitude of fold change in the protein's expression pattern. Solid and dashed lines represent direct and indirect interactions, respectively. For further details on symbols and relationships, see http://qiagen.force.com/KnowledgeBase/articles/Basic_Technical_Q_A/Legend. Inset table (**B**): Top canonical pathways and their corresponding molecular targets associated with the network shown. Z-scores indicate activation or suppression of the corresponding pathway (positive or negative values, respectively; N/A: no prediction can be made).

(**A**)

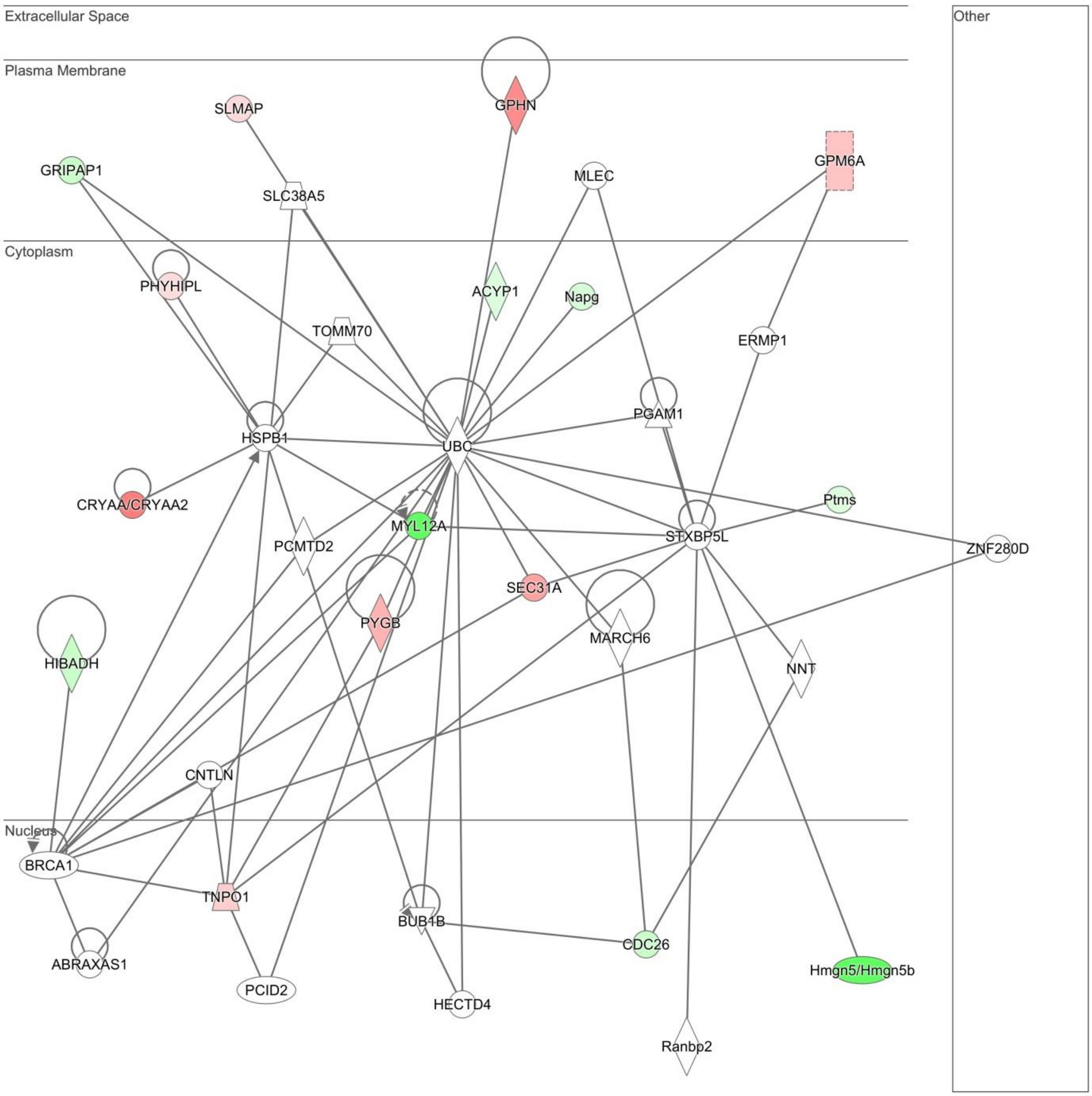

(**B**)

**Canonical pathway**	**Targets**	**Z-score**	***p*-value**
Protein ubiquitination pathway	BRCA1, CRYAA/CRYAA2, HSPB1, UBC	N/A	2.86E-06
Sirtuin signaling pathway	PGAM1, TOMM70	-0.3	3.26E-07
Protein kinase A signaling	CDC26, MYL12A, PYGB	2.3	6.03E-03

Figure S11. IPA® network linked to gene expression, RNA damage and repair, RNA post transcriptional modification (**A**). The network was assembled from E_2-regulated proteins in the retina of OVX rats through identification by label-free shotgun proteomics. In the network, red and green colors denote upregulation and downregulation in response to E2 treatment, respectively. The intensity of color indicates the relative magnitude of fold change in the protein's expression pattern. Solid and dashed lines represent direct and indirect interactions, respectively. For further details on symbols and relationships, see http://qiagen.force.com/KnowledgeBase/articles/Basic_Technical_Q_A/Legend. Inset table (**B**): Top canonical pathways and their corresponding molecular targets associated with the network shown. Z-scores indicate activation or suppression of the corresponding pathway (positive or negative values, respectively; N/A: no prediction can be made).

(**A**)

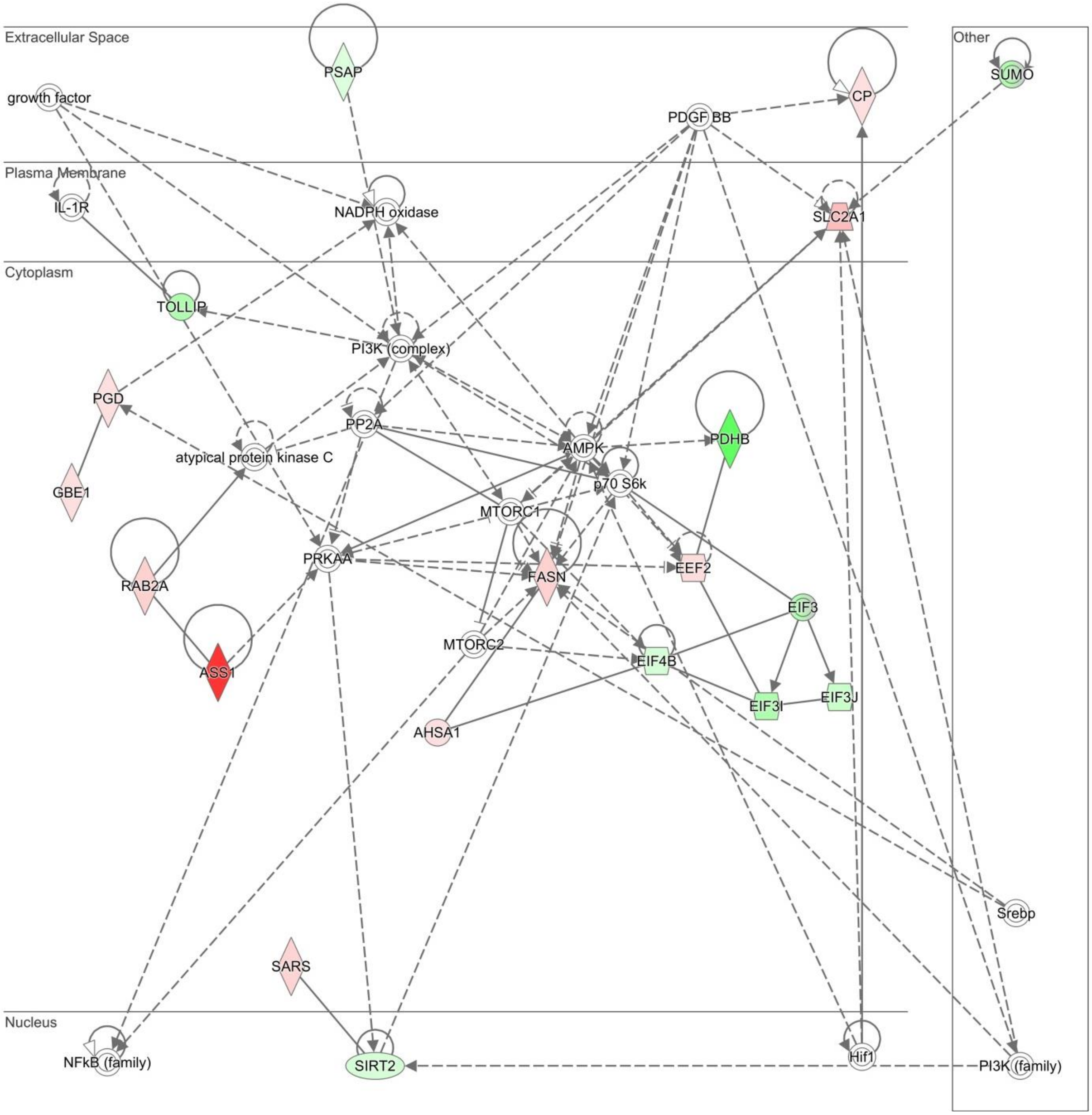

(**B**)

Canonical pathway	Targets	Z-score	*p*-value
mTOR signaling	AMPK, Atypical protein kinase C, EIF3, EIF31, EIF3J, EIF4B, Growth factor, Hif1, MTORC1, MTORC2, p70 S6k, PI3K, PI3K (family), PP2A, PRKAA	1.3	1.25E-02
Sirtuin signaling pathway	AMPK, Hif1, MtorC1, Mtor C2, NFkB, PRKAA, SIRT2, SLC2A1, Srebp	-0.3	3.26E-07
AMPK pathway	AMPK, EEF2, FASN, MTORC1, MTORC2, p70 S6k, PI3K, P13K (family), PP2A, PRKAA, RAB2A, SLC2A1	1.9	3.09E-04

Figure S12. IPA® network linked to nucleic acid metabolism, protein synthesis, small-molecule biochemistry (**A**). The network was assembled from E_2-regulated proteins in the retina of OVX rats through identification by label-free shotgun proteomics. In the network, red and green colors denote upregulation and downregulation in response to E2 treatment, respectively. The intensity of color indicates the relative magnitude of fold change in the protein's expression pattern. Solid and dashed lines represent direct and indirect interactions, respectively. For further details on symbols and relationships, see http://qiagen.force.com/KnowledgeBase/articles/Basic_Technical_Q_A/Legend. Inset table (**B**): Top canonical pathways and their corresponding molecular targets associated with the network shown. Z-scores indicate activation or suppression of the corresponding pathway (positive or negative values, respectively).

(**A**)

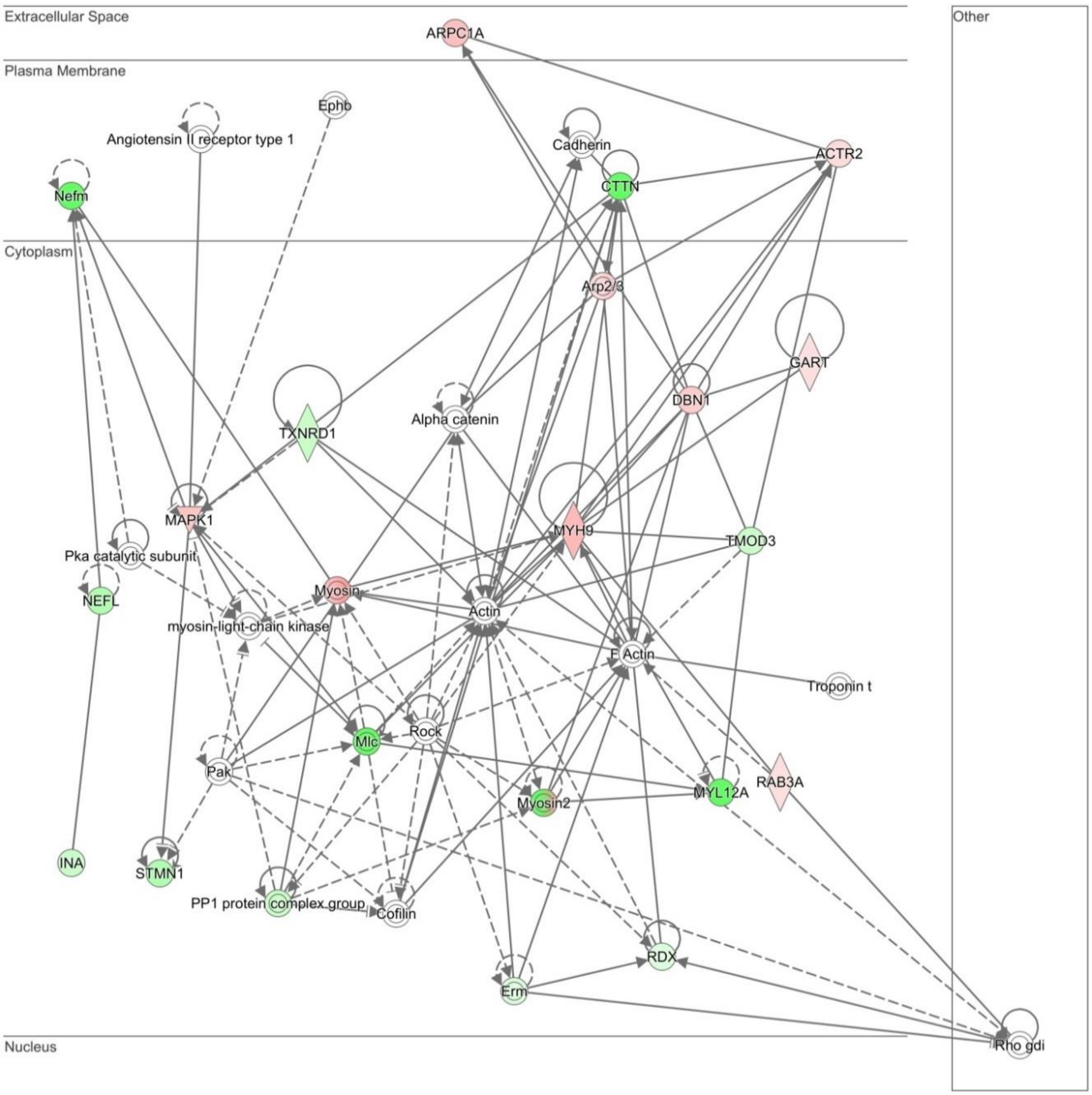

(**B**)

**Canonical pathway**	**Targets**	**Z-score**	***p*-value**
Actin cytoskeleton signaling	Actin, ACTR2, Arp2/3, ARPC1A, Cofilin, Erm, F Actin, MAPK1, Mlc, MYH9, MYL12A, Myosin2, Myosin, myosine-light-chain kinase, Pak, PP1 protein complex group, RDX, Rock	1.7	9.96E-05
Axonal guidance signaling	ACTR2, Arp2/3, ARPC1A, Cofilin, Ephb, MAPK1, Pak, Pka catalytic subunit, Mlc, MYL12A, Myosin2, Myosin, Rock	N/A	1.76E-01
Synaptogenesis signaling pathway	ACTR2, Arp2/3, ARPC1A, Cadherin, Cofilin, Ephb, MAPK1, Pak, Pka catalytic subunit, RAB3A	1.1	2.20E-04

Figure S13. IPA® network linked to cellular assembly and organization, cellular function and maintenance, and tissue development (**A**). The network was assembled from E_2-regulated proteins in the retina of OVX rats through identification by label-free shotgun proteomics. In the network, red and green colors denote upregulation and downregulation in response to E2 treatment, respectively. The intensity of color indicates the relative magnitude of fold change in the protein's expression pattern. Solid and dashed lines represent direct and indirect interactions, respectively. For further details on symbols and relationships, see http://qiagen.force.com/KnowledgeBase/articles/Basic_Technical_Q_A/Legend. Inset table (**B**): Top canonical pathways and their corresponding molecular targets associated with the network shown. Z-scores indicate activation or suppression of the corresponding pathway (positive or negative values, respectively; N/A: no prediction can be made).

(**A**)

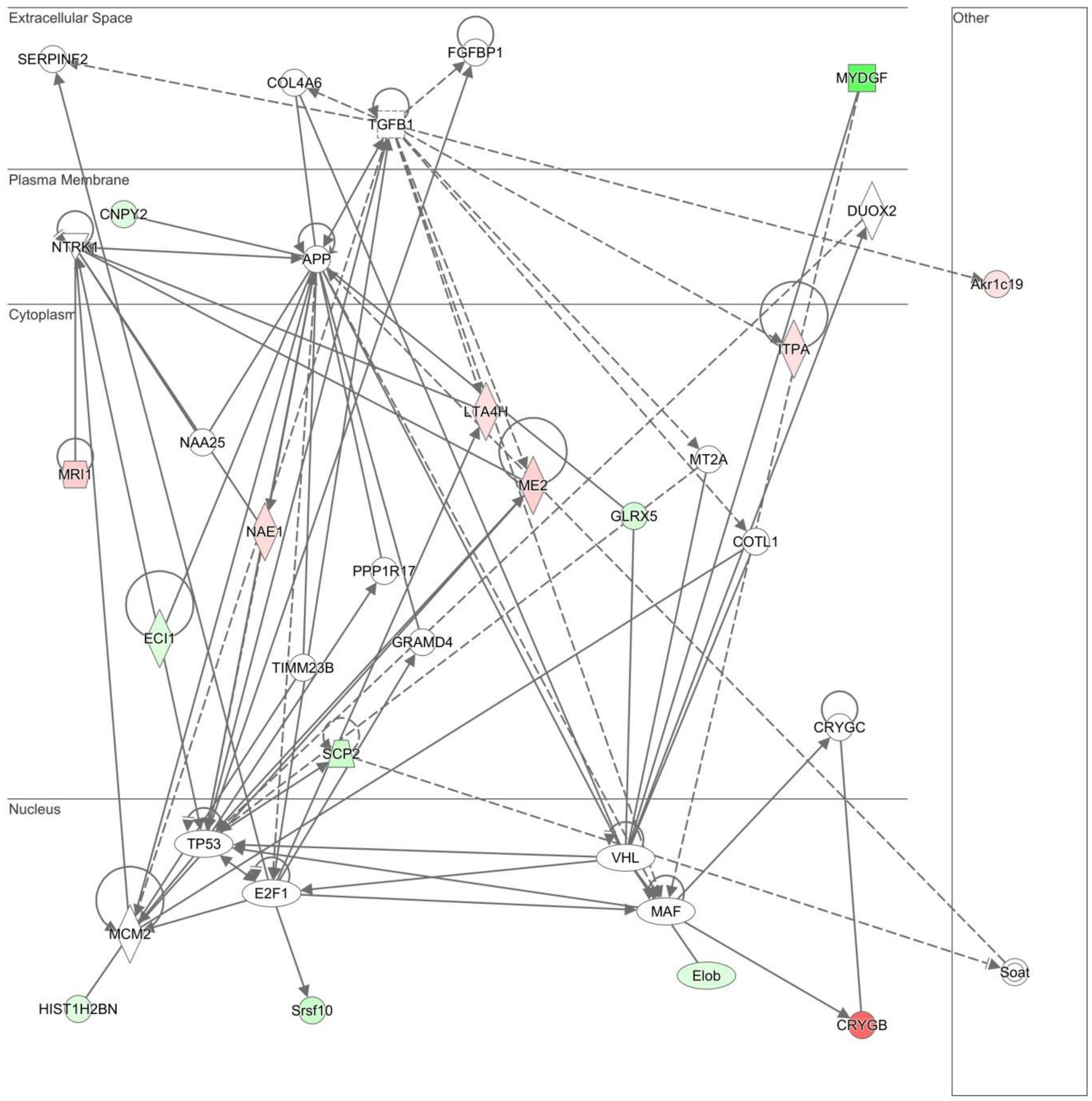

(**B**)

Canonical pathway	Targets	Z-score	*p*-value
Sirtuin signaling pathway	APP, E2F1, TIMM23B, TP53	-0.3	3.26E-07
Aryl hydrocarbon receptor signaling	E2F1, TGFB1, TP53	N/A	

Figure S14. IPA® network linked to cell morphology, cellular compromise, cellular function and maintenance (**A**). The network was assembled from E_2-regulated proteins in the retina of OVX rats through identification by label-free shotgun proteomics. In the network, red and green colors denote upregulation and downregulation in response to E2 treatment, respectively. The intensity of color indicates the relative magnitude of fold change in the protein's expression pattern. Solid and dashed lines represent direct and indirect interactions, respectively. For further details on symbols and relationships, see http://qiagen.force.com/KnowledgeBase/articles/Basic_Technical_Q_A/Legend. Inset table (**B**): Top canonical pathways and their corresponding molecular targets associated with the network shown. Z-scores indicate activation or suppression of the corresponding pathway (positive or negative values, respectively; N/A: no prediction can be made).

(**A**)

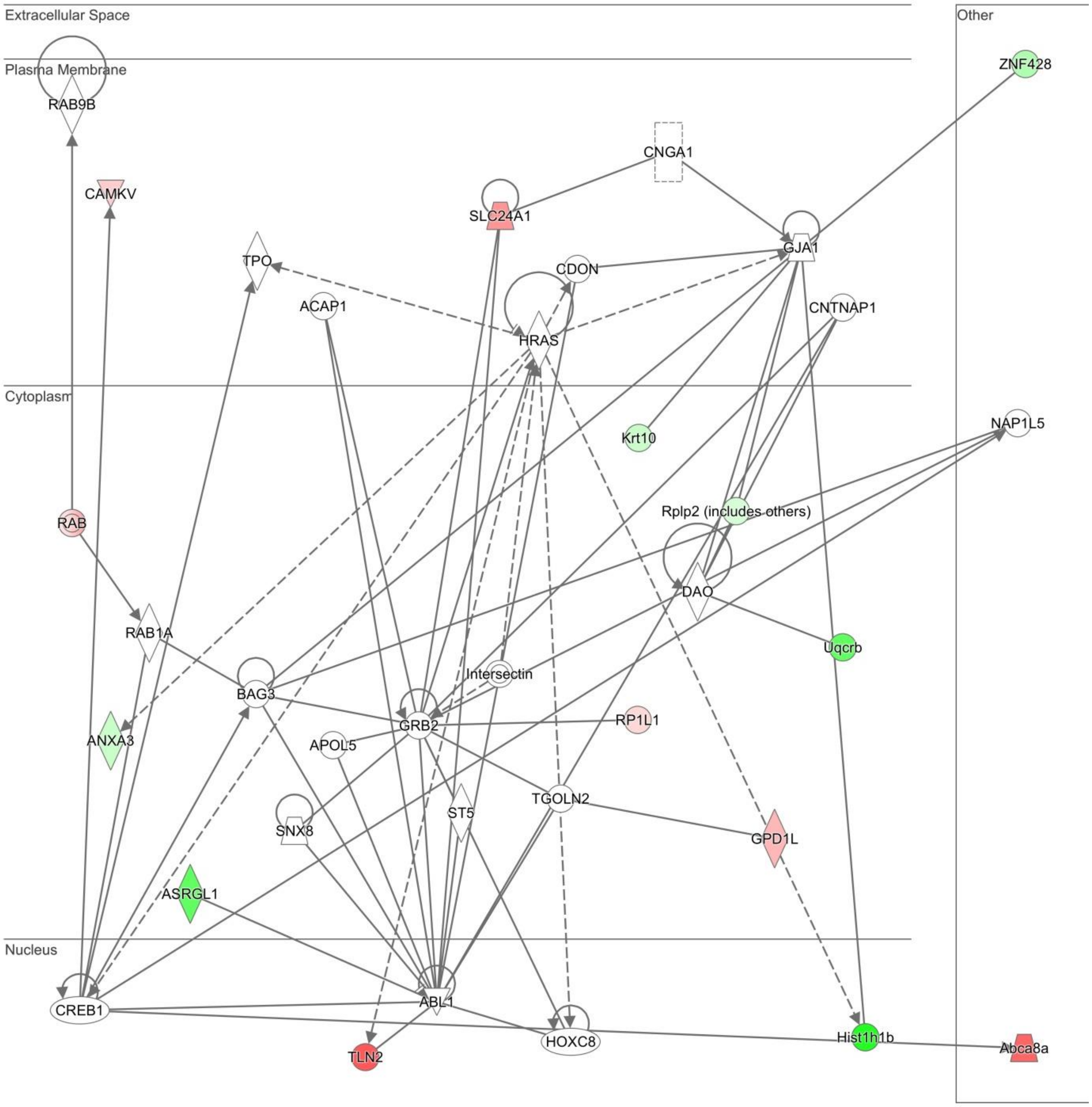

(**B**)

**Canonical pathway**	**Targets**	**Z-score**	***p*-value**
ERK/MAPK signaling	CREB1, GRB2, HRAS, TLN2	1.8	2.40E-2
Axonal guidance signaling	ALB1, GRB2, HRAS, Intersectin	N/A	1.76E-01
Synaptogenesis signaling pathway	CNTNAP1, CREB1, GRB2, HRAS, Intersectin, RAB	1.1	2.20E-04

Figure S15. IPA® network linked to cancer, cellular growth and proliferation, organismal injury and abnormalities (**A**). The network was assembled from E_2-regulated proteins in the retina of OVX rats through identification by label-free shotgun proteomics. In the network, red and green colors denote upregulation and downregulation in response to E2 treatment, respectively. The intensity of color indicates the relative magnitude of fold change in the protein's expression pattern. Solid and dashed lines represent direct and indirect interactions, respectively. For further details on symbols and relationships, see http://qiagen.force.com/KnowledgeBase/articles/Basic_Technical_Q_A/Legend. Inset table (**B**): Top canonical pathways and their corresponding molecular targets associated with the network shown. Z-scores indicate activation or suppression of the corresponding pathway (positive or negative values, respectively; N/A: no prediction can be made).

(**A**)

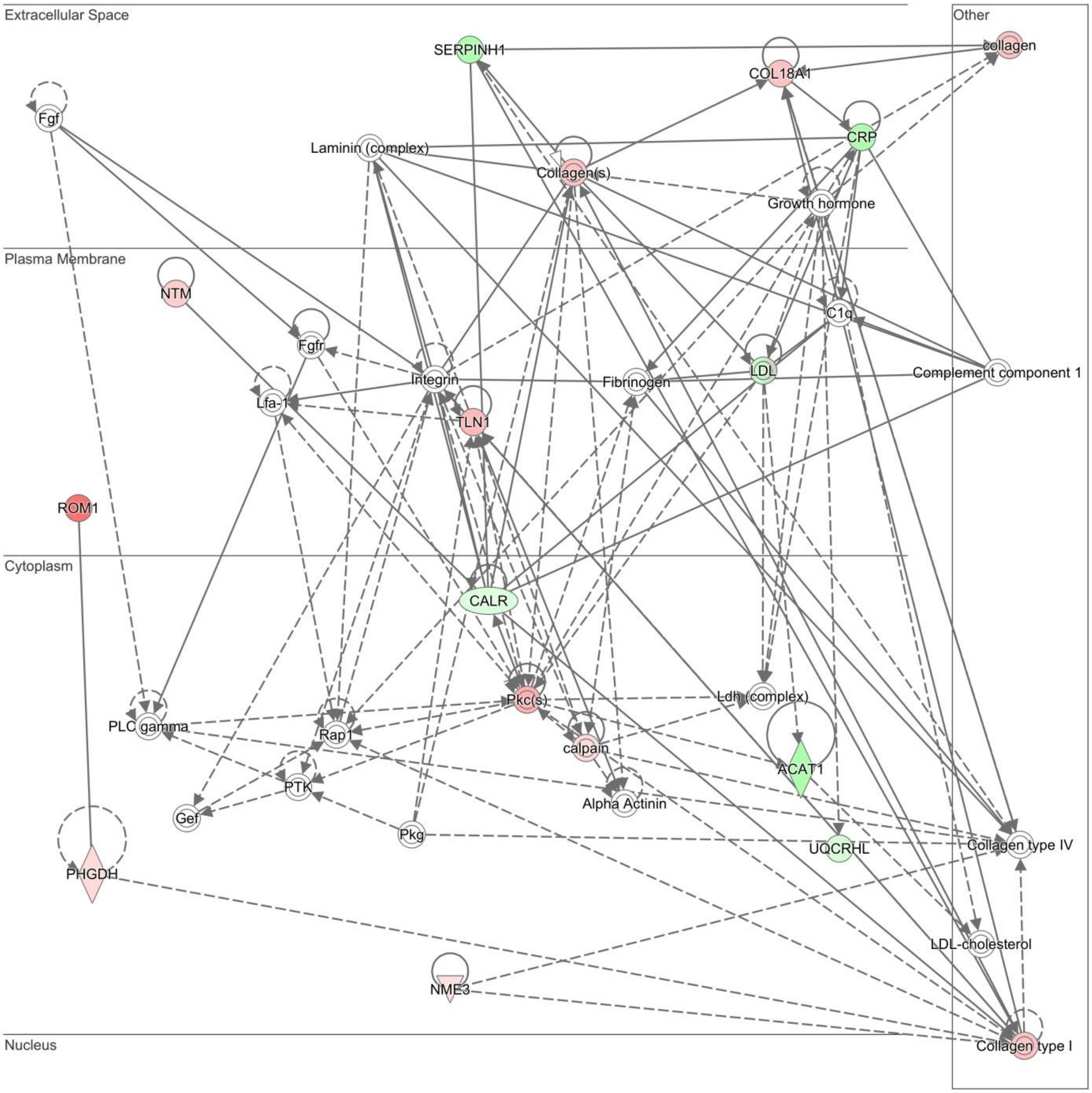

(**B**)

Canonical pathway	Targets	Z-score	*p*-value
Actin cytoskeleton signaling	Alpha Actinin, Fgfr, Gef, Integrin, Lfa-1, PTK, Rap1, TLN1	1.7	9.96E-05
Axonal guidance signaling	Gef, Integrin, Lfa-1, Pkc, PLC gamma, PTK, Rap1	N/A	1.76E-01
ERK/MAPK signaling	Integrin, Lfa-1, Pkc, PLC gamma, PTK, Rap1, TLN1	1.8	2.40E-2

Figure S16. IPA® network linked to amino acid metabolism, cellular assembly and organization, small- molecule biochemistry (**A**). The network was assembled from E_2-regulated proteins in the retina of OVX rats through identification by label-free shotgun proteomics. In the network, red and green colors denote upregulation and downregulation in response to E2 treatment, respectively. The intensity of color indicates the relative magnitude of fold change in the protein's expression pattern. Solid and dashed lines represent direct and indirect interactions, respectively. For further details on symbols and relationships, see http://qiagen.force.com/KnowledgeBase/articles/Basic_Technical_Q_A/Legend. Inset table (**B**): Top canonical pathways and their corresponding molecular targets associated with the network shown. Z-scores indicate activation or suppression of the corresponding pathway (positive or negative values, respectively; N/A: no prediction can be made).

(A)

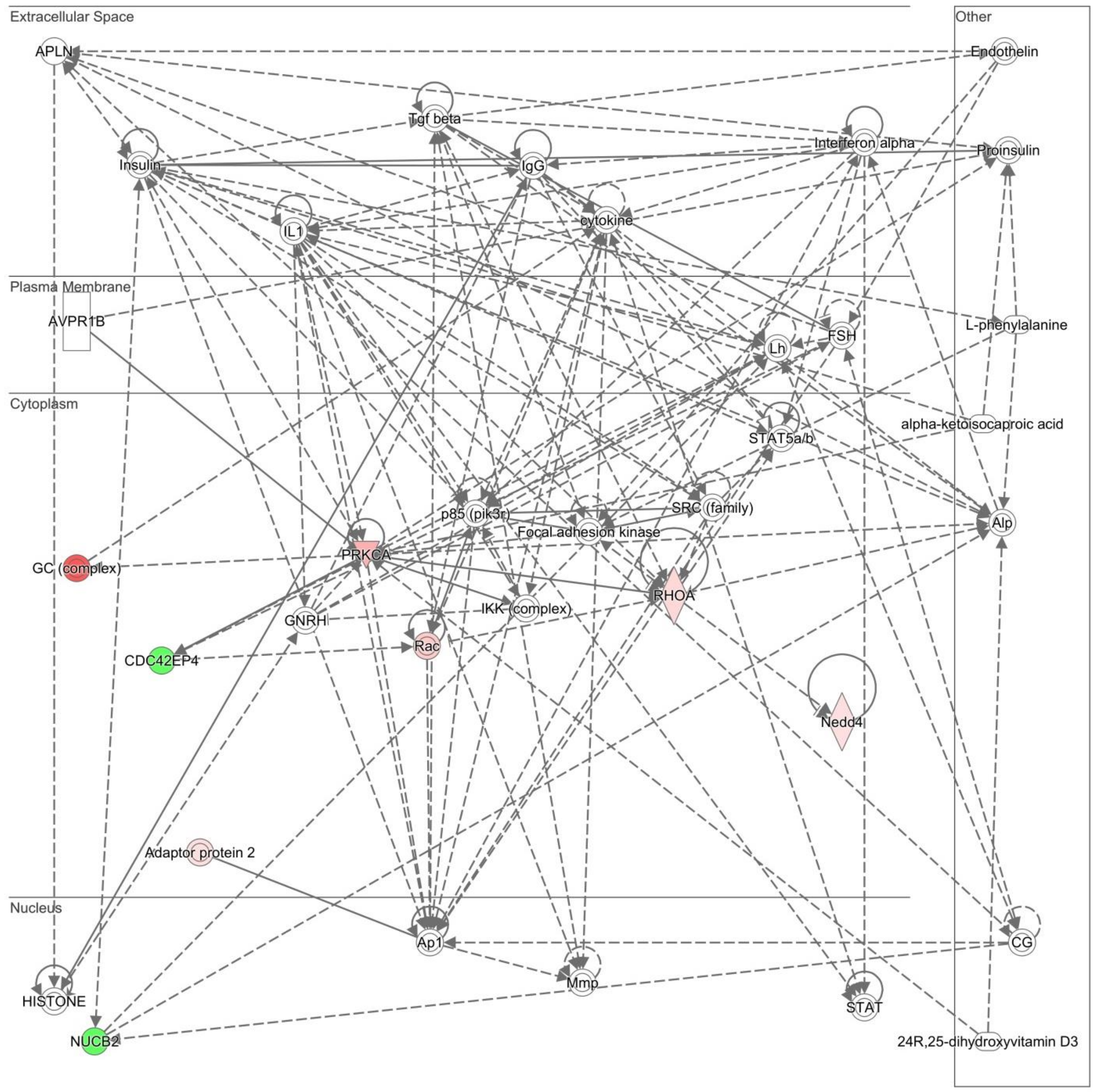

© 2000-2019 QIAGEN. All rights reserved.

(B)

**Canonical pathway**	**Targets**	**Z-score**	***p*-value**
Axonal guidance signaling	Focal adhesion kinase, Mmp, p85 (pik3r), PRKCA, Rac, RHOA, SRC	N/A	1.76E-01
ERK/MAPK signaling	Ap1, Focal adhesion kinase, HISTONE, p85 (pik3r), PRKCA, Rac, SRC, STAT	1.8	2.40E-02

Figure S17. IPA® network linked to cell death and survival, cellular development, cellular growth and proliferation (**A**). The network was assembled from E_2-regulated proteins in the retina of OVX rats through identification by label-free shotgun proteomics. In the network, red and green colors denote upregulation and downregulation in response to E2 treatment, respectively. The intensity of color indicates the relative magnitude of fold change in the protein's expression pattern. Solid and dashed lines represent direct and indirect interactions, respectively. For further details on symbols and relationships, see http://qiagen.force.com/KnowledgeBase/articles/Basic_Technical_Q_A/Legend. Inset table (**B**): Top canonical pathways and their corresponding molecular targets associated with the network shown. Z-scores indicate activation or suppression of the corresponding pathway (positive or negative values, respectively; N/A: no prediction can be made).

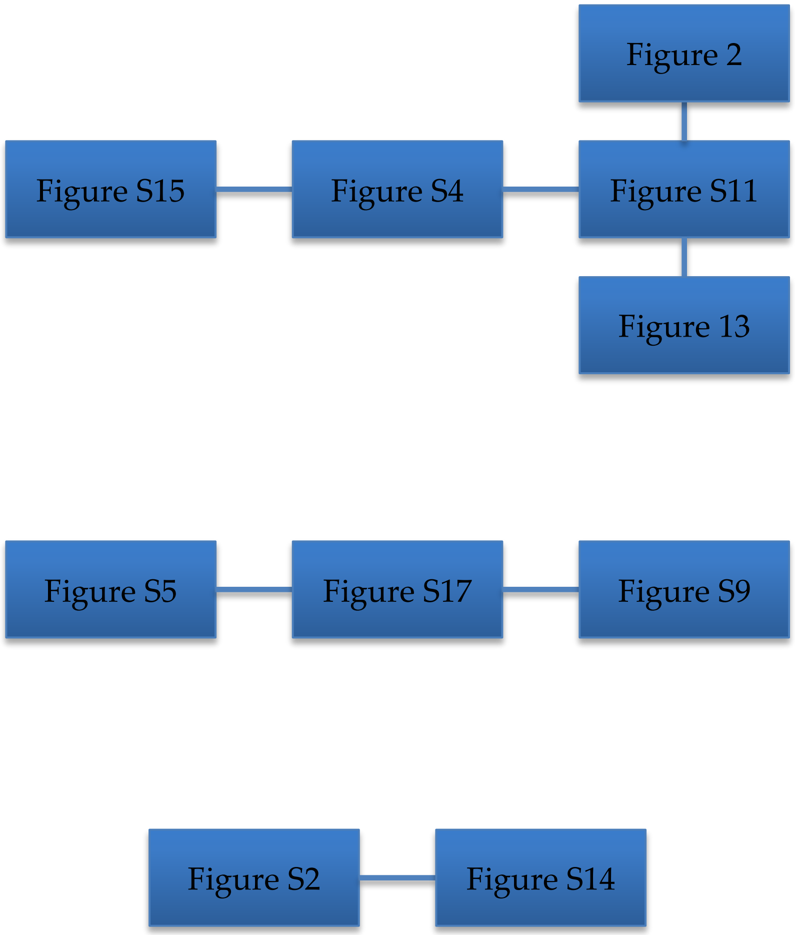

Figure S18. Overlapping IPA® networks.
